# Ran GTPase-Activating Protein 1 Is a Therapeutic Target in Diffuse Large B-Cell Lymphoma

**DOI:** 10.1371/journal.pone.0079863

**Published:** 2013-11-06

**Authors:** Kung-Chao Chang, Wei-Chao Chang, Yao Chang, Liang-Yi Hung, Chien-Hsien Lai, Yu-Min Yeh, Yu-Wei Chou, Chung-Hsuan Chen

**Affiliations:** 1 Department of Pathology, College of Medicine, National Cheng Kung University and Hospital, Tainan, Taiwan; 2 Genomics Research Center, Academia Sinica, Taipei, Taiwan; 3 Division of Infectious Diseases, National Health Research Institute, Tainan, Taiwan; 4 Institute of Bioinformatics and Biosignal Transduction, National Cheng Kung University, Tainan, Taiwan; 5 Department of Internal Medicine, College of Medicine, National Cheng Kung University and Hospital, Tainan, Taiwan,; University of Nebraska - Lincoln, United States of America

## Abstract

Lymphoma-specific biomarkers contribute to therapeutic strategies and the study of tumorigenesis. Diffuse large B-cell lymphoma (DLBCL) is the most common type of malignant lymphoma. However, only 50% of patients experience long-term survival after current treatment; therefore, developing novel therapeutic strategies is warranted. Comparative proteomic analysis of two DLBCL lines with a B-lymphoblastoid cell line (LCL) showed differential expression of Ran GTPase-activating protein 1 (RanGAP1) between them, which was confirmed using immunoblotting. Immunostaining showed that the majority of DLBCLs (92%, 46/50) were RanGAP1^+^, while reactive lymphoid hyperplasia (n = 12) was RanGAP1^+^ predominantly in germinal centers. RanGAP1 was also highly expressed in other B-cell lymphomas (BCL, n = 180) with brisk mitotic activity (B-lymphoblastic lymphoma/leukemia: 93%, and Burkitt lymphoma: 95%) or cell-cycle dysregulation (mantle cell lymphoma: 83%, and Hodgkin’s lymphoma 91%). Interestingly, serum RanGAP1 level was higher in patients with high-grade BCL (1.71 ± 2.28 ng/mL, n = 62) than in low-grade BCL (0.75 ± 2.12 ng/mL, n = 52) and healthy controls (0.55 ± 1.58 ng/mL, n = 75) (high-grade BCL vs. low-grade BCL, p = 0.002; high-grade BCL vs. control, p < 0.001, Mann-Whitney U test). *In vitro*, RNA interference of RanGAP1 showed no effect on LCL but enhanced DLBCL cell death (41% vs. 60%; p = 0.035) and cell-cycle arrest (G0/G1: 39% vs. 49%, G2/M: 19.0% vs. 7.5%; p = 0.030) along with decreased expression of TPX2 and Aurora kinases, the central regulators of mitotic cell division. Furthermore, ON 01910.Na (Estybon), a multikinase inhibitor induced cell death, mitotic cell arrest, and hyperphosphorylation of RanGAP1 in DLBCL cell lines but no effects in normal B and T cells. Therefore, RanGAP1 is a promising marker and therapeutic target for aggressive B-cell lymphoma, especially DLBCL.

## Introduction

Tumor biomarkers are pivotal for screening, diagnosing, and following-up cancers. Lymphoma-specific markers also contribute to treatment strategy, prognostic stratification, and the study of tumorigenesis. Current clinically useful biomarkers for lymphoma management are both scarce and non-specific. For example, serum lactate dehydrogenase (LDH) is a widely used biomarker in lymphoma patients and is linked to prognosis [1]. However, its low specificity limits its clinical application because, in addition to tumor progression, an elevated LDH level is also found in other non-neoplastic conditions, such as myocardial damage [2]. Moreover, LDH provides no insight into tumor biology [3]. Serum beta-2 microglobulin, an established prognostic factor for multiple myeloma, has also been used for non-Hodgkin’s lymphoma patients as a prognostic factor [4]. Similarly, its low specificity and sometimes low sensitivity diminish its clinical utility [5].

Several serum biomarkers for lymphomas have been suggested as being clinically useful: cystatin C [6], soluble intercellular adhesion molecule-1 (s-ICAM-1/s-CD54) [7], soluble Fas/CD95/APO-1 [8], soluble tumor necrosis factor receptor 2 (sTNF-R2) [9], soluble interleukin-2 receptor (sIL-2R) [10], nm23-H1 protein [11], and soluble CD44 [12]. However, none of these markers is specific for detecting lymphomas because they are also elevated in other cancers and even in non-neoplastic diseases [13-15]. Thus, all the markers emphasize prognostic correlation rather than lymphoma treatment or insights into tumorigenesis.

Diffuse large B-cell lymphoma (DLBCL) is the most common subtype of non-Hodgkin’s lymphoma and accounts for 30-40% of all lymphoma cases worldwide [16,17]. The mainstay strategy for treating DLBCL is multidrug immunochemotherapy. However, long-time survival is achieved in only 50% of patients, which underscores the need to develop innovative therapeutic strategies [17,18]. The present study used the comparative proteomics approach to search for candidate lymphoma biomarkers as the proper targets for treatment and study of lymphoma biology. 

## Materials and Methods

### Culturing DLBCL and B-lymphoblastoid cell lines

Two DLBCL cell lines HT (ACC 567) and SU-DHL-5 (ACC 571) were purchased from DSMZ (Braunschweig, Germany). For comparison, we used a B-lymphoblastoid cell line (LCL), which was derived from human blood B cells immortalized by Epstein-Barr virus infection [19,20]. The culture protocol is described in Supporting Materials and Methods in File S1.

### Proteomic analysis

The procedures were done as previously described [21,22], and consisted of three steps: protein separation and in-gel digestion, LC LTQ-FT ICR MS analysis, and Mascot search and label-free quantitative analysis. Proteomic analysis was done in duplicate. The details are provided in Supporting Materials and Methods in File S1.

### Immunoblotting assay

Cell lysates were lysed in 1X Radio-Immunoprecipitation Assay (RIPA) sample buffer (Upstate Biotechnology, Lake Placid, NY, USA) containing 50 mM Tris-HCl (pH 8.8) with protease inhibitor cocktail added (Roche Applied Science, Indianapolis, IN, USA). Differential subcellular fractions were separated into cytosol and nucleus using a protein extraction kit (ProteoExtract Subcellular Proteome Extraction Kit; EMD Biosciences, Inc., La Jolla, CA, USA). Polyacrylamide gel electrophoresis and immunodetection of Ran GTPase-activating protein 1 (RanGAP1, 1:1000, C-5, sc-28322; Santa Cruz Biotechnology, Santa Cruz, CA, USA) and phospho-RanGAP1 (pSer428, 1:1000, R5280; Sigma-Aldrich, Inc., St. Louis, OM, USA) were done as previously described [23]. The ratio was expressed as the amount of RanGAP1 divided by the corresponding amount of GAPDH (glyceraldehyde 3-phosphate dehydrogenase, 1:5000, 6C5, sc-32233; Santa Cruz) using an imaging analyzer (White Light Transilluminator; Bio-Rad Laboratories, Hercules, CA, USA). Other antibodies for Western blots included Aurora-A (1:4000, 35C1, GTX13824; GeneTex, Irvine, CA, USA), Aurora-B (1:4000, A5102, polyclonal; Sigma-Aldrich, St. Louis, MO, USA), TPX2 (1:1000, 3164C6a, sc-81413; Santa Cruz), INCENP (inner centromere protein, 1:500, H-153, sc-67175; Santa Cruz), α-tubulin as a cytosol marker (Ab-2; 1:5000, DM1A; NeoMarkers, Fremont, CA, USA), and histone H1 as a nuclear marker (AE-4; 1:1000; Millipore Corporation, Billerica, MA, USA). Immunoblotting was done in duplicate.

### Immunofluorescent staining

After cytospinning the cells and fixing them in acetone, the slides containing DLBCL cells and LCL were washed with phosphate buffer solution (PBS, pH 7.4), and then incubated with primary antibodies against RanGAP1 (1:100, C-5, sc-28322; Santa Cruz) for 2 hours at room temperature in the dark. After the cells had been washed with PBS, they were incubated with dye-labeled secondary antibodies. Nuclear DNA was stained with 4'-6-diamidino-2-phenylindole (DAPI; 1:1000; Invitrogen, Carlsbad, CA, USA).

### Immunohistochemical staining

Immunohistochemical staining was done on deparaffinized tissue sections of formalin-fixed material after microwave-enhanced epitope retrieval as previously described [24]. Staining intensity recognizable in a low-power field (×40) for more than 30% of the tumor cells was deemed positive. The primary antibody, RanGAP1 was from Santa Cruz Biotechnology (1:100, C-5, sc-28322; Santa Cruz).

The cases enrolled for RanGAP1 staining consisted of primary DLBCL (n = 50), lymphoid hyperplasia (n = 12), and other B-cell lymphomas (BCL, n = 180) from the archival files at National Cheng Kung University Hospital. The germinal center (GC) vs. activated B-cell immunophenotype and Ki-67 (MIB-1) proliferation index were determined for DLBCL as described previously [24,25]. Double staining of Ki-67 and RanGAP1 was performed with an automated stainer (Bond-Max; Leica Biosystems Melbourne Pty Ltd, Melbourne, Australia).

### Enzyme-linked immunosorbent assay (ELISA) for RanGAP1

To determine whether RanGAP1 reflects the disease status, the serum level of RanGAP1 was measured using ELISA for DLBCL patients at diagnosis. A colorimetric noncompetitive (immunometric sandwich assay) ELISA was done; procedure details are provided in Supporting Materials and Methods in File S1.

Serum from high-grade BCL (n = 62: Burkitt lymphoma, n = 12; DLBCL, n = 50), low-grade BCL (n = 52: follicular lymphoma, n = 27; mucosa-associated lymphoid tissue (MALT) type lymphoma, n = 14; small lymphocytic lymphoma, n = 4; nodal marginal zone lymphoma, n = 3; splenic marginal zone lymphoma, n = 2; lymphoplasmacytic lymphoma, n = 2) and healthy controls (n = 75) were enrolled for RanGAP1 ELISA. Clinical data—gender, age, serum level of LDH, tumor site, B symptoms, Ann Arbor stage, IPI (international prognostic index) score, treatment modality, and overall survival in months—were obtained by reviewing patient charts. All DLBCL patients were followed up and treated with a curative CHOP or R-CHOP (rituximab, cyclophosphamide, doxorubicin, vincristine, and prednisone) regimen. For selected patients, surgical intervention or radiotherapy preceded chemotherapy.

### Transfecting RANGAP1-specific shRNA into cell lines

Short hairpin RNAs (shRNAs) were designed against the target sequence of RANGAP1 (5′-CAAGAGTGAAGACAAGGTCAA-3′, bases 1834-1854, NM_002883.2). Other two sets of small interfering RNA (siRNA) for RANGAP1 knockdown were also performed in duplicate. The sequences and detailed procedures are described in Supporting Materials and Methods in File S1. The inhibition of RanGAP1 expression was evaluated using immunoblotting. The cell lines were cultured and then collected for further analysis 48 h after they had been transfected.

### Cell death and cell cycle assays by flow cytometry

Apoptosis and other forms of cell death were evaluated by measuring the DNA content using annexin V and propidium iodide (PI) affinity as previously described [26]. Briefly, each sample of 2.6 × 10^6^ cells was transfected with RANGAP1-specific shRNA (shRANGAP1) or control vector, and then cultured in 6 ml of medium. Each sample of 1.5 ml was collected after 48 h. The samples were then centrifuged, and the pellet was incubated with staining solution (PI [50 μg/ml]; 0.1% sodium citrate; 0.1% triton) overnight at 4°C in the dark. Core DNA content was measured using a logarithmic amplification in the FL2 (for annexin V) and FL3 (for PI) channels of the flow cytometer (FACSCalibur with CellQuest Pro 4.0.2; Becton Dickinson, Franklin Lakes, NJ, USA) [27].

Cell-cycle analysis was also measured using flow cytometry. The distribution of the DNA content of individual cells was stained with PI and measured with CellQuest Pro 4.0.2 using a linear amplification in the FL3 channel.

### Quantitative real-time polymerase chain reaction (Q-PCR)

The Q-PCR assay was done as previously described [28]. Briefly, total RNA was isolated using an RNA extraction kit (TRIzol; Invitrogen, Carlsbad, CA, USA). Three micrograms of RNA was used to generate cDNA with reverse transcriptase (SuperScript III; Invitrogen). The primers used for Q-PCR are described in Supporting Materials and Methods in File S1.

### Assessment of ON 01910.Na cytotoxic effects on DLBCL and LCL

The cytotoxic effects of ON01910.Na (Estybon/Novonex/Rigosertib, Cat No. S1362, Selleckchem.com, Houston, TX, USA) on LCL and DLBCL cell lines (HT and SU-DHL-5) were first assessed by the Cell Counting Kit-8 (CCK-8) MTT assay (Sigma-Aldrich, cat.96992). LCL (8×10^4^/100 μl) and DLBCL (4×10^4^/100 μl) cells were cultured in 96-well microplates. Cells were then treated with ON01910.Na at different concentrations from 0.016, 0.031, 0.063, 0.125, 0.25, to 0.5 μM for 2 days. Afterward, 100 µl serum free medium containing 20 µl CCK-8 solutions was added to each well and incubated for 1.5 hours at 37°C. The absorbance was determined with a microplate reader at 450 nm. After determination of the lethal dose 50 (LD50), cell death and cell cycle assays were done by flow cytometry as previously. Each assay was repeated in triplicate.

### Statistical analysis

Appropriate statistical tests—*t*-test, Kendall tau (τ) correlation coefficient, and Mann-Whitney U tests—were used to examine the relationships and correlations between variables. Overall survival was measured from the initial diagnosis until death from any cause; follow-up data of surviving patients were assessed at the last contact date. Estimates of overall survival distribution were calculated using the Kaplan and Meier method [29]. Time-to-event distributions were compared using the log-rank test [30]. The analyses were done using SPSS 13.0 (SPSS, Inc., Chicago, IL, USA).

### Ethics Statement

 The study was approved by our institutional review board (Institutional Review Board, National Cheng Kung University Hospital-HR-95-72) and was done in accord with the Helsinki Declaration of 1975 as revised in 1983. The written consent was given by the patients for their information to be stored in the hospital database and used for research.

## Results

### Proteomic analysis yielded 20 proteins overexpressed in DLBCL cell lines compared with the B-lymphoblastoid cell line

Two DLBCL cell lines (HT and SU-DHL-5) and a B-lymphoblastoid cell line (LCL) were used for comparison to search for candidate biomarkers from cell lysates. Eighty-nine proteins were identified in total with 20 proteins up-expressed and 69 proteins down-expressed in tumor cell lines. These 20 highly-expressed proteins are listed in Table 1 and Table S1 in File S1. We chose RanGAP1 as the interesting candidate for further studies because of its dual subcellular localization. Besides, RanGAP1 is a key regulator of the Ran GTP/GDP cycle and a mitosis coordinator [31,32].

**Table 1:** Proteins more highly expressed in DLBCL cell lines (HT and SU) than in the B-LCL determined using comparative proteomic analysis.

**Uniprot Acc#**	**Protein Name**	**Location**	**Signal intensity (average)**			**Ratio**	
			**HT**	**SU**	**LCL**	**HT/LCL**	**SU/LCL**
P23526	Adenosylhomocysteinase	Cytoplasm	4484400	4518400	3144300	1.43	1.44
P27707	Deoxycytidine kinase	Nucleus	638620	497930	79610	8.02	6.25
P52701	DNA mismatch repair protein Msh6	Nucleus	0	5932900	807720	0	7.35
P49736	DNA replication licensing factor MCM2	Nucleus	6241500	10184000	4821100	1.29	2.11
P25205	DNA replication licensing factor MCM3	Nucleus	5105500	5689800	3205400	1.59	1.78
P33991	DNA replication licensing factor MCM4	Nucleus	8135000	14964000	5048900	1.61	2.96
P33992	DNA replication licensing factor MCM5	Nucleus	4448300	9623600	2634800	1.69	3.65
Q14566	DNA replication licensing factor MCM6	Nucleus	8205700	9524200	3819200	2.15	2.49
P33993	DNA replication licensing factor MCM7	Nucleus	7250500	15046000	5793800	1.25	2.6
C9J4C3	DNA topoisomerase 2	Nucleus	5475900	13128000	1458800	3.75	9
Q02880	DNA topoisomerase 2-beta	Nucleus	2538400	952320	626520	4.05	1.52
P62807	Histone H2B type 1-C/E/F/G/I	Nucleus	85579000	162440000	129050000	0.66	1.26
P36776	Lon protease homolog, mitochondrial	Mitochondria	2233600	7393900	2772700	0.81	2.67
Q14676	Mediator of DNA damage checkpoint protein 1	Nucleus	6142000	1894700	1541300	3.98	1.23
P19338	Nucleolin, isoform 1	Nucleus	102890000	90123000	58881000	1.75	1.53
P13010	Protein X-ray repair cross-complementing protein 5	Nucleus	22411000	22996000	19606000	1.14	1.17
**P46060**	**Ran GTPase-activating protein 1**	**Cytoplasm & nucleus**	**1220100**	**1154900**	**911260**	**1.34**	**1.27**
P43487	Ran-specific GTPase-activating protein	Nucleus	5070100	2663300	3566000	1.42	0.75
P23246	Splicing factor, proline- and glutamine-rich	Nucleus	41996000	35759000	22272000	1.89	1.61
Q13428	Treacle protein, isoform 2	Nucleus	9425700	14198000	6420200	1.47	2.21

Abbreviations: DLBCL, diffuse large B-cell lymphoma; B-LCL, B-lymphoblastoid cell line.

### Western blotting confirmed higher RanGAP1 expression in DLBCL lines

**Figure 1:**
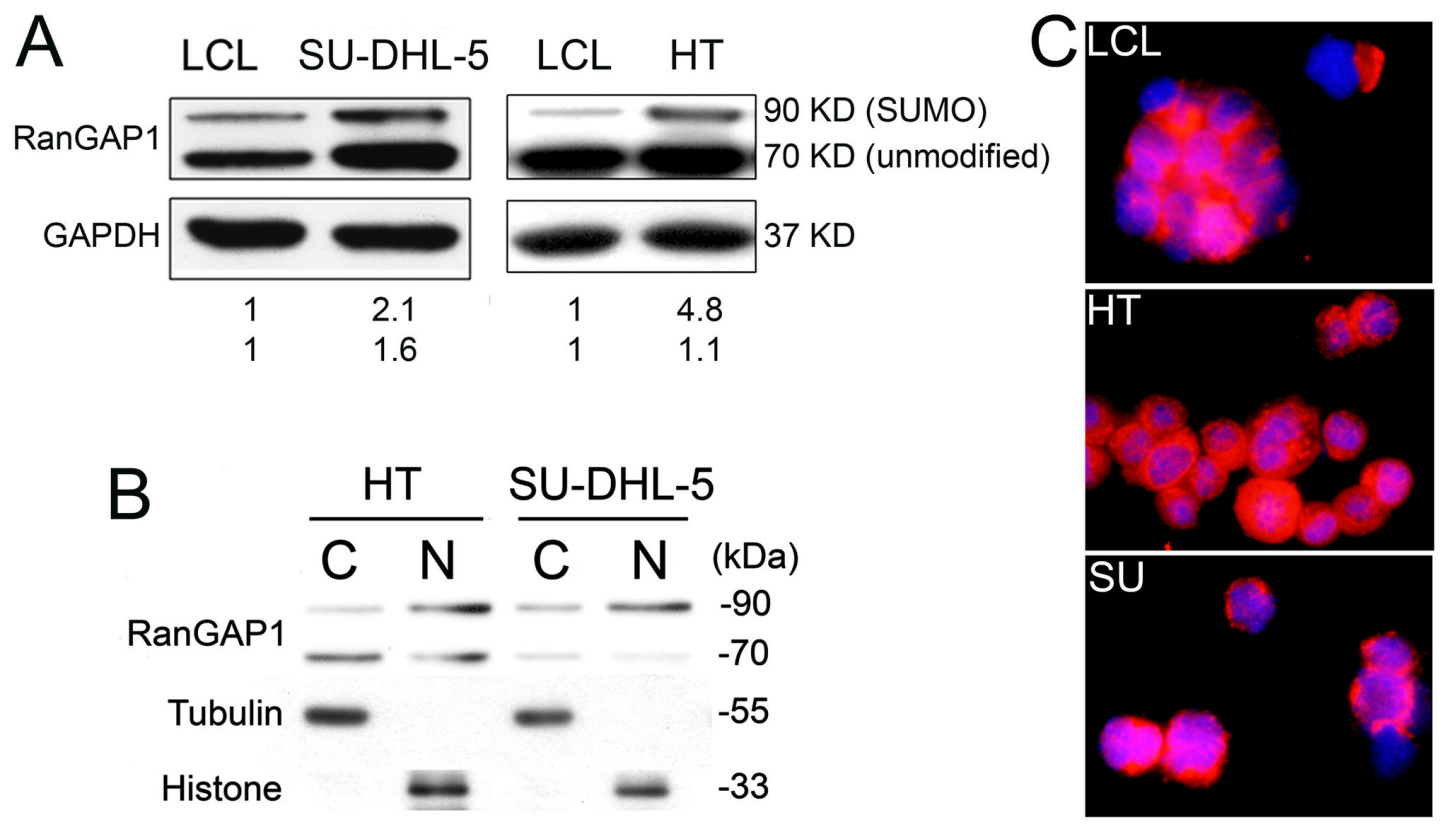
RanGAP1 expression in DLBCL and LCL cell lines. (**A**) Western blotting shows higher RanGAP1 expression in the SU-DHL-5 cell line than in the LCL (lymphoblastoid cell line) (1.6:1, p = 0.035; unmodified form, 70 kDa) and (2.1:1, p = 0.045; SUMO-1 modified form, 90 kDa). HT shows a similar result (1.1:1, unmodified form; 4.8:1, SUMO-1 form). (**B**) Differential subcellular fractions show RanGAP1 present in both cytoplasm and nucleus. (**C**) Immunofluorescence highlights the localization of RanGAP1 in both cytosol and perinucleus with intranuclear dot-like distribution (red: RanGAP1; blue: DAPI; merged: purple).

Western blotting was used to compare the expression levels of RanGAP1 on neoplastic and reactive B cells. SU-DHL-5 cells expressed 1.6 times (unmodified form, 70 kDa) and 2.1 times (small ubiquitin-related modifier [SUMO]-1 modified form, 90 kDa) more RanGAP1 than did the LCL cells (Figure 1A). HT cells showed a similar result (1.1 and 4.8 times for unmodified and SUMO form, respectively, Figure 1A). Differential subcellular fractions showed RanGAP1 present in both cytoplasm and nucleus (Figure 1B). Immunofluorescence demonstrated the cytoplasmic and perinuclear localization (Figure 1C) as described previously [33].

### RanGAP1 immunohistochemically stained the majority of DLBCL cases but only germinal centers of lymph nodes

**Figure 2:**
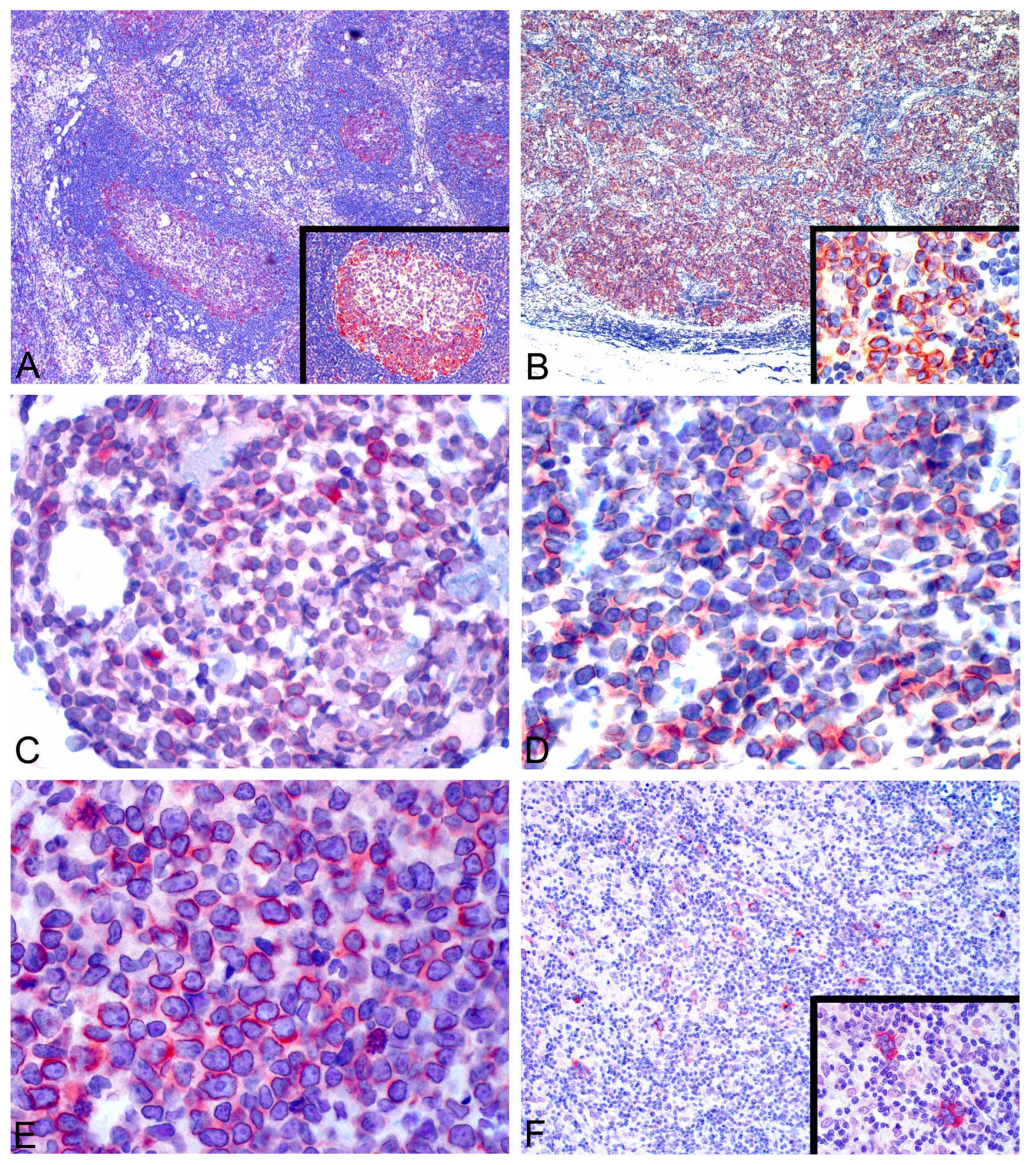
RanGAP1 staining in reactive hyperplasia and B-cell lymphomas. (**A**, ×40) In hyperplastic lymph nodes, RanGAP1 expression is predominantly in the peripheral rim of germinal centers, with occasional positivity in dark zones (**inset**, ×100). (**B**, ×40) DLBCL cells show both cytoplasmic and perinuclear staining (**inset**, ×400) in the majority of tumor cells. The expression of RanGAP1 is also frequently found in B-lymphoblastic lymphoma/leukemia (**C**, ×400), Burkitt lymphoma (**D**, ×400), mantle cell lymphoma (**E**, ×400), and Hodgkin’s lymphoma (**F**, ×100**; lower** **inset**, ×400). Perinuclear accentuation is evident in B-lymphoblastic lymphoma/leukemia (**C**), Burkitt lymphoma (**D**) and mantle cell lymphoma (**E**). Images were photographed using a digital microscope camera (DP12; Olympus Co., Tokyo, Japan) and processed using Adobe Photoshop 7.0 (Adobe Systems, San Jose, CA, USA).

Immunohistochemical staining of nodal hyperplasia cases (n = 12) showed RanGAP1 positivity mainly in germinal centers with occasionally in dark zone only (Figure 2A). Scattered histiocytes in interfollicular areas were also positive, but the other cells, including T cells, were negative. In contrast, the majority of DLBCL cases (46/50, 92%) were positive for RanGAP1 staining in perinuclear and cytoplasmic regions (Figure 2B).

### Expression of RanGAP1 in aggressive BCL and Hodgkin’s lymphoma

Other BCL (n = 180, Table 2) were immunostained with RanGAP1. Interestingly, RanGAP1 was frequently expressed in tumors with brisk mitotic activity (B-lymphoblastic lymphoma/leukemia: 93.3% [Figure 2C], and Burkitt lymphoma: 94.6% [Figure 2D]) or with cell-cycle aberrations (mantle cell lymphoma: 83.3% [Figure 2E], and Hodgkin’s lymphoma: 90.5% [Figure 2F]). Other types of BCL showed rare or uncommon expression of RanGAP1, including small lymphocytic lymphoma (6.3%), follicular lymphoma (23.5%), marginal zone lymphoma, MALT type (16.7%), and lymphoplasmacytic lymphoma (36.4%).

**Table 2:** Results of RanGAP1 immunostaining in B-cell lymphomas.

**Lymphoma Types**	**No.**	**Positivity**	**(%)**	**Gender M/F**	**Age (mean)**
Diffuse large B-cell lymphoma	50	46/50	92	24/26	60.6
**Other B-cell lymphomas**	**180**				
B-lymphoblastic lymphoma/leukemia	15	14/15	93.3	10/5	34.9
Burkitt lymphoma	37	35/37	94.6	25/12	6.0
Mantle cell lymphoma	12	10/12	83.3	11/1	67.3
Hodgkin lymphoma	42	38/42	90.5	34/8	6.7
Small lymphocytic lymphoma	16	1/16	6.3	12/4	64.9
Follicular lymphoma	17	4/17	23.5	9/8	56.9
Marginal zone lymphoma, MALT type	30	5/30	16.7	13/17	60.1
Lymphoplasmacytic lymphoma	11	4/11	36.4	9/2	73.5

Abbreviations: MALT, mucosa-associated lymphoid tissue.

### Higher serum level of RanGAP1 in patients with high-grade BCL than in low-grade BCL and healthy controls

Since there is no commercial assay for RanGAP1, the colorimetric noncompetitive (immunometric sandwich) ELISA has been established using two different anti-RanGAP1 antibodies (RanGAP1 C-5 and RanGAP1 N-19; Santa Cruz) with different antigen-binding sites. The former was raised against amino acids 408-587 and the latter against a peptide mapping at the N-terminus of RanGAP1 of human origin. Preliminary experiments showed that the detection range was 0-20 ng/mL (Figure S1 in File S1). The ELISA signal was not influenced by the presence of lipid. ELISA showed relatively high levels of RanGAP1 in the conditioned media of SU-DHL-5 cells (4.51 ng/mL) compared with LCL cells (1.09 ng/mL). For clinical samples, serum levels of RanGAP1 were higher in patients with high-grade BCL (1.71 ± 2.28 ng/mL, n = 62) than in low-grade BCL (0.75 ± 2.12 ng/mL, n = 52) and healthy controls (0.55 ± 1.58 ng/mL, n = 75) (high-grade BCL vs. low-grade BCL, p = 0.002; high-grade BCL vs. control, p < 0.001, Mann-Whitney U test, Figure S2 in File S1). However, the RanGAP1 serum level was not so sensitive, since half (n = 31) cases of high-grade BCL were not elevated. 

### RanGAP1 had no prognostic significance for patients with DLBCL

We next evaluated the correlation between RanGAP1 serum level and other clinicopathologic factors in DLBCL cases: tumor stage, treatment response, and patient survival. In our DLBCL cohort, there was positive correlation between high IPI score (≥ 3) and B symptoms (Kendall tau (τ) correlation coefficient: 0.260, *p* = 0.041), between high LDH level and B symptoms (τ correlation coefficient: 0.247, *p* = 0.039), and high stage disease (τ correlation coefficient: 0.389, *p* < 0.001). On survival analyses (Table 3), the poor prognostic factors were old age (*p* = 0.001), B symptoms (*p* = 0.009), and a high IPI score (*p* = 0.003). RanGAP1 serum level had no prognostic significance and showed no correlation with other clinicopathologic factors, including the GC immunophenotype and a high Ki-67 index (> 80%).

**Table 3:** Clinicopathologic factors affecting survival of patients with DLBCL.

**Parameter**	**Worst factor**	**No. (%)**	**p-value (log rank test)**
Gender	Male	23 (46)	0.151
Age (years)	Old (> 60)	30 (60)	0.001
B symptoms	Present	16 (32)	0.009
Location	Nodal vs. extranodal	23 (46)	0.881
LDH	> 200 IU/L	35 (70)	0.443
Phenotype	Non-GC type	14 (28)	0.694
Ki-67 index	> 80%	23 (46)	0.497
IPI score	3-5	22 (44)	0.003
Stage	3-4	29 (58)	0.154
RanGAP1	> 2.57 ng/mL	31 (62)	0.660

Abbreviations: LDH, lactate dehydrogenase; IPI, international prognosis index; RanGAP1, Ran GTPase-activating protein 1; GC, germinal center.

### Knockdown of RanGAP1 mRNA increased DLBCL cell death and cell cycle arrest but had no effect on non-neoplastic LCL cells

**Figure 3:**
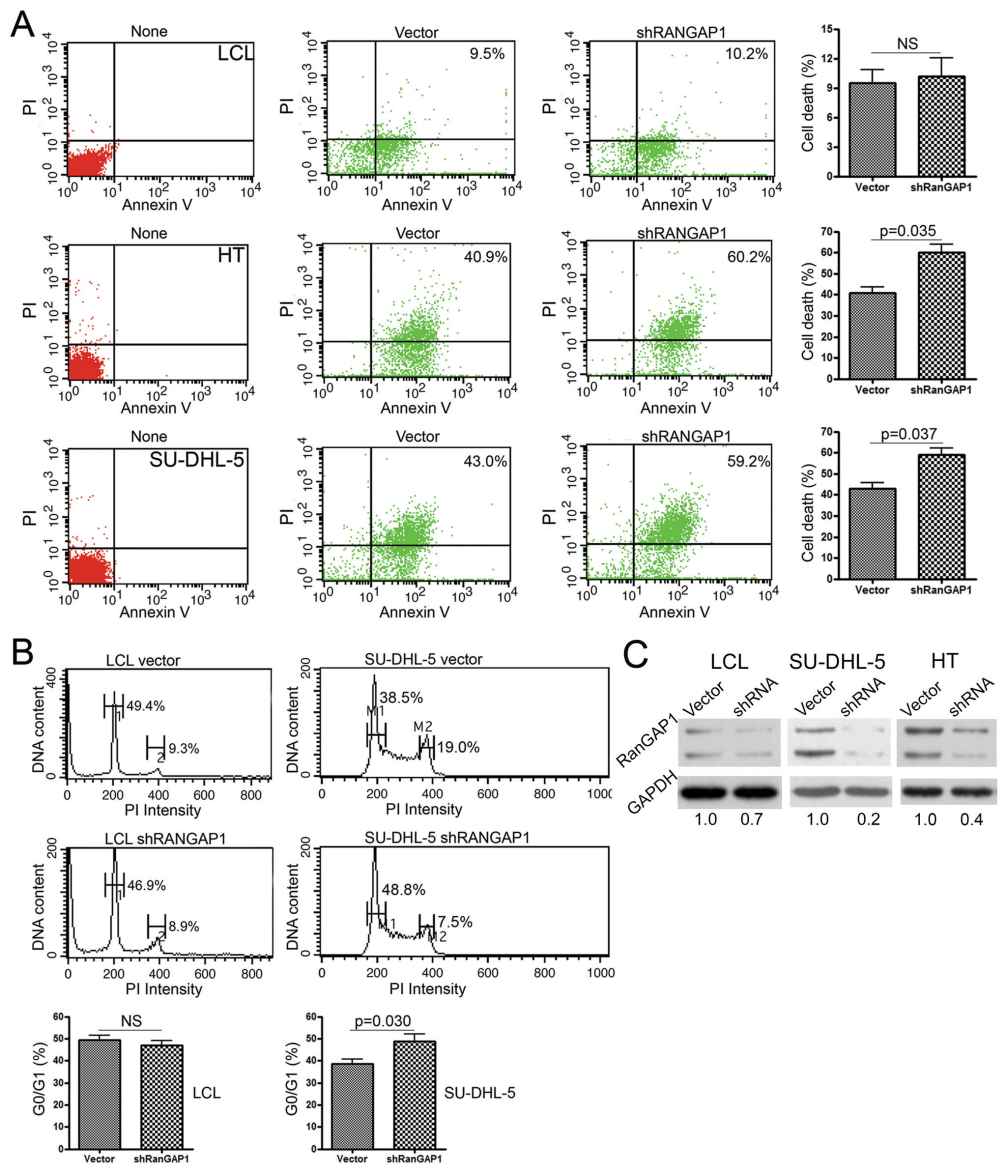
RanGAP1 RNA interference increased tumor cell death and cell-cycle arrest but had no effect on non-neoplastic LCL cells. (**A**) After transfection, the effect of inhibiting RanGAP1 was evaluated in the LCL (upper panel), HT (middle panel), and SU-DHL-5 cell lines (lower panel) for cell death, measured using annexin V and PI (propidium iodide). LCL cells show no difference in cell apoptosis between control vector (9.5%) and shRANGAP1 (RANGAP1-specific shRNA) (10.2%; NS, not significant). In contrast, apoptosis was higher in the HT (vector, 40.9% vs. shRANGAP1, 60.2%, p = 0.035) and SU-DHL-5 cell lines (vector, 43.0% vs. shRANGAP1, 59.2%, p = 0.037). None: non-transfected maternal cells. (**B**) Cell-cycle analysis shows no effect on LCL (left panel, 1: G0/G1, vector, 49.4% vs. shRANGAP1, 46.9%; 2: G2/M, vector, 9.3% vs. shRANGAP1, 8.9%; NS, not significant), but it does show G0/G1 cell-cycle arrest in SU-DHL-5 cells (right panel, M1: G0/G1, vector, 38.5% vs. shRANGAP1, 48.8%; M2: G2/M, vector, 19.0% vs. shRANGAP1, 7.5%, p = 0.030). (**C**) Western blotting shows a marked decrease (vector, 1.0 vs. shRANGAP1, 0.2 with GAPDH normalization) of RanGAP1 expression in SU-DHL-5 and HT (vector, 1.0 vs. shRANGAP1, 0.4) after RNA interference of RANGAP1 by shRNA.

To test the function of RanGAP1 protein, we knocked down RanGAP1 mRNA to see the effects on cell survival and the cell cycle in DLBCL and LCL cells. The transfection rates for each cell line were as follows: LCL: 41-43%; SU-DHL-5: 76-86%; HT: 51-60%. In contrast to no effect on LCL (9.5% [vector] vs. 10.2% [shRANGAP1]) (Figure 3A, upper panel), HT showed significantly (*p* = 0.035, paired *t*-test) tumor cell death (40.9% [vector] vs. 60.2% [shRANGAP1]) (Figure 3A, middle panel), as did the SU-DHL-5 cell line (*p* = 0.037; 43.0% [vector] vs. 59.2% [shRANGAP1]) (Figure 3A, lower panel). Besides, RNA interference of RanGAP1 had no effect on the cell cycle of LCL cells (G0/G1: 49.4% [vector] vs. 46.9% [shRANGAP1]; G2/M: 9.3% [vector] vs. 8.9% [shRANGAP1]) (Figure 3B, left panel), but it significantly (*p* = 0.030, paired *t*-test) induced G0/G1 cell-cycle arrest in SU-DHL-5 cells (G0/G1: 38.5% [vector] vs. 48.8% [shRANGAP1]; G2/M: 19.0% [vector] vs. 7.5% [shRANGAP1]) (Figure 3B, right panel). shRNA clearly inhibited RanGAP1 expression (Figure 3C).

### RanGAP1 knockdown reduced expression of Aurora kinases and TPX2 in DLBCL lines

**Figure 4:**
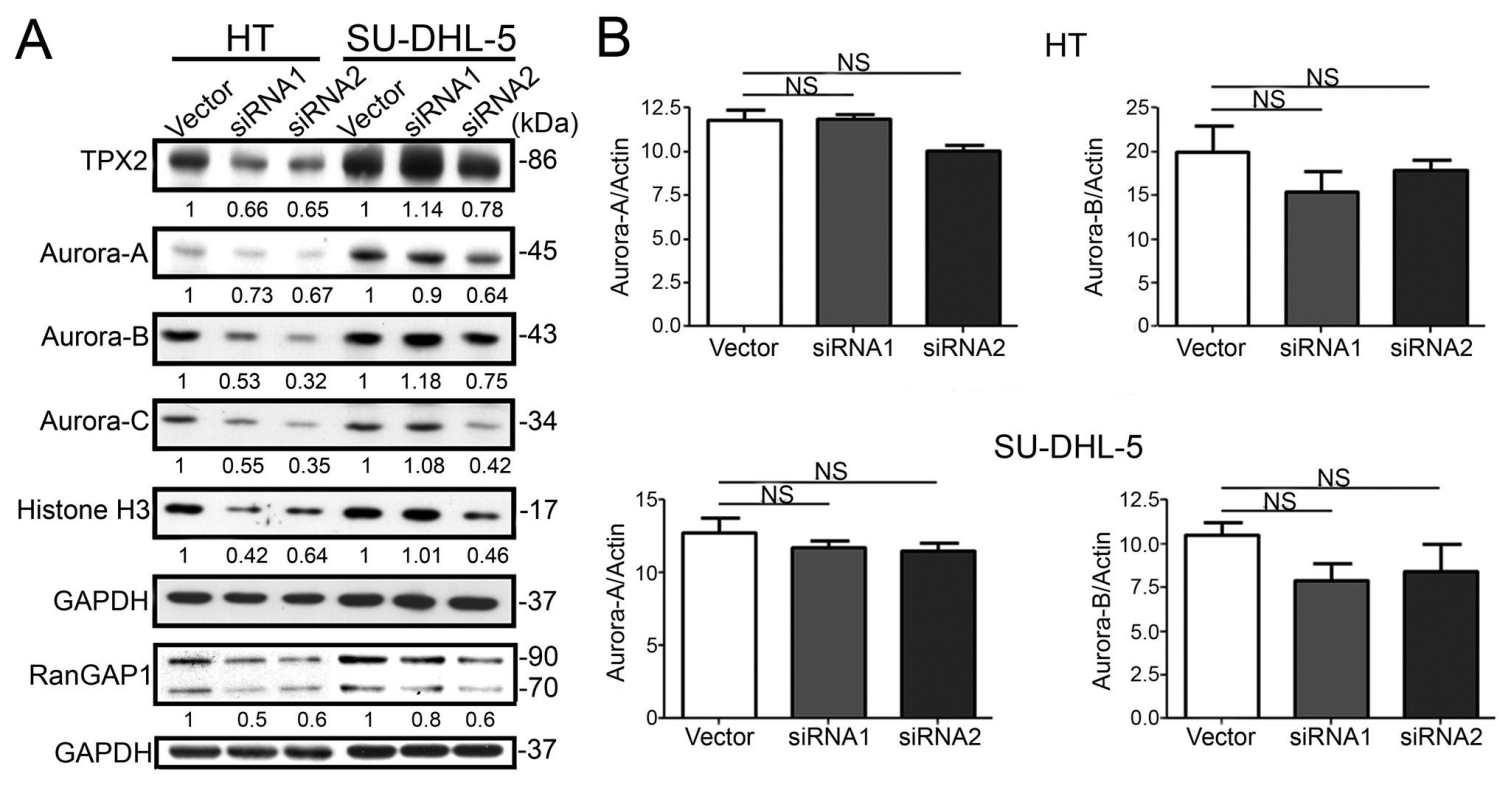
RanGAP1 knockdown inhibited the expression of Aurora kinases and TPX2, but did not affect their transcription. (**A**) Thirty micrograms of total cell lysates from RanGAP1 siRNA transfected cells (two sets of siRNA sequences, siRNA1 and siRNA2) were harvested and subjected to immunoblotting analysis as indicated in the figures. After normalization, RanGAP1 knockdown inhibited the expression of the Aurora-A, -B, and -C kinases, and TPX2. The inhibition was more effective on HT cells and by siRNA2 sequence. (**B**) Q-PCR shows no significant (NS) decrease in mRNA level of Aurora-A and Aurora-B kinases in DLBCL cells, HT cells (upper panel), or SU-DHL-5 cells (lower panel). The bar graph shows the means ± SD of three experiments.

To decipher the mechanism underlying RanGAP1-knockdown-induced cell-cycle arrest and tumor cell death in DLBCL lines, we tested the effects of RanGAP1 siRNA on the expression of Aurora kinases and TPX2. The former are key regulators of mitotic cell division [34], and the latter is central in spindle assembly [35]. The RanGAP1-specific siRNAs (siRNA1 and siRNA2) downregulated the expression of TPX2 and Aurora-A, -B, and -C kinases in DLBCL lines (Figure 4A). Q-PCR analysis showed that there was no significant decrease in the mRNA levels of Aurora kinases (Figure 4B). These data indicated that the RanGAP1-specific siRNAs inhibited the expression of Aurora kinases but did not affect kinase transcription.

### ON 01910.Na induced cell death, mitotic cell arrest and hyperphosphorylation of RanGAP1 in DLBCL cell lines but mild effects in non-neoplastic LCL

**Figure 5:**
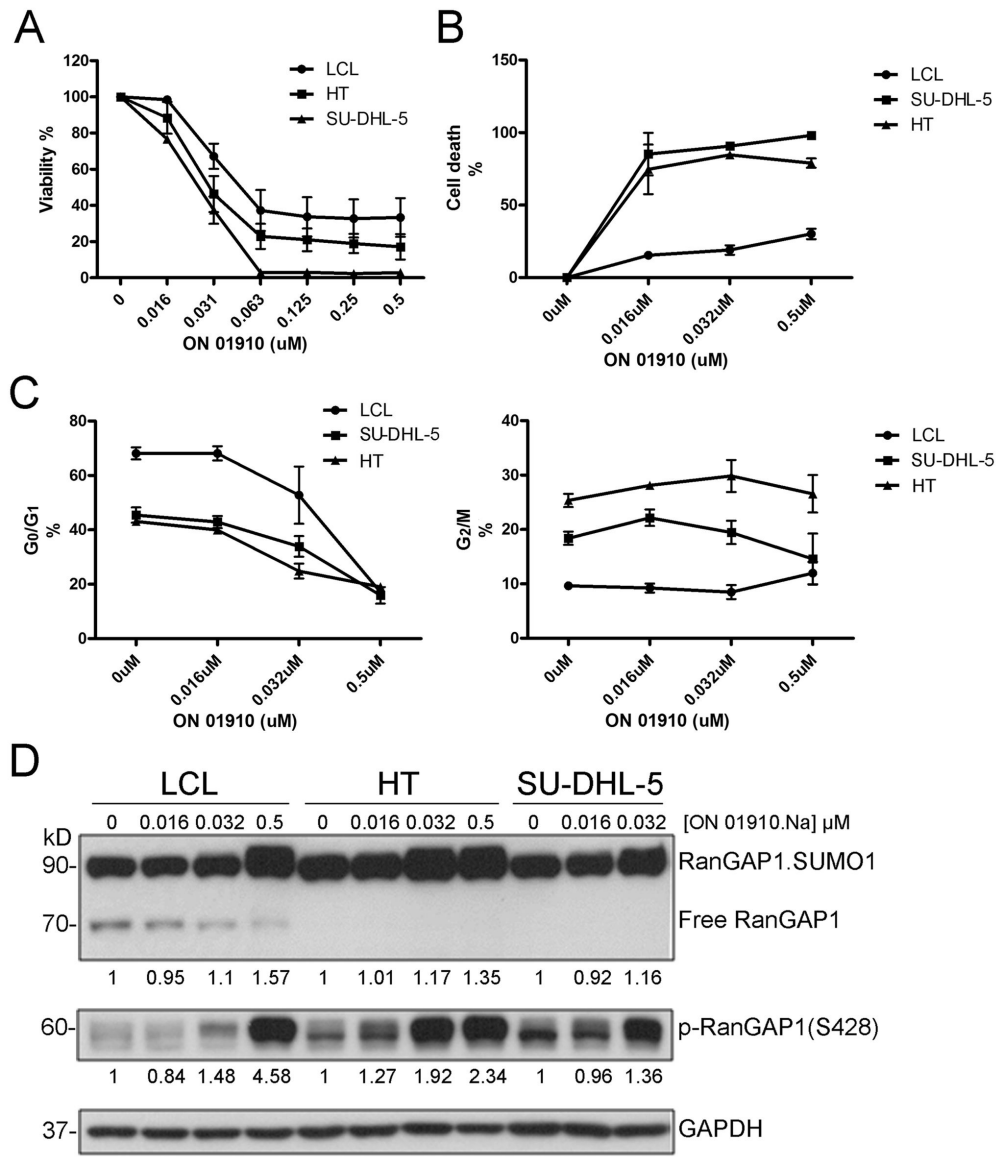
ON 01910.Na induced more cell death, mitotic cell arrest and hyperphosphorylation of RanGAP1 in DLBCL cell lines (HT and SU-DHL-5) than in non-neoplastic LCL. (**A**) MTT assay shows the ID50 of ON 01910.Na is around 0.031 μM for DLBCL lines. (**B**) ON 01910.Na shows relatively selective cytotoxicity to DLBCL by flow cytometry analysis. (**C**) ON 01910.Na induces more evident mitotic cell arrest (G2/M, right panel) for DLBCL lines at the concentration between 0.016 and 0.032 μM on cell cycle analysis with flow cytometry. (**D**) Along with cell death, immunoblotting shows ON 01910.Na induces hyperphosphorylation of RanGAP1, increased expression of RanGAP1.SUMO1 but decreased expression of free unmodified RanGAP1. No viable SU-DHL-5 cells were available for immunoblotting at 0.5 μM of ON 01910.Na.

The ID50 of ON 01910.Na was around 0.031 μM for DLBCL lines by the MTT assay (Figure 5A). ON 01910.Na showed relatively selective cytotoxicity to DLBCL by flow cytometry analysis (Figure 5B), and induced more evident mitotic cell arrest in DLBCL lines than in LCL at the concentration between 0.016 and 0.032 μM on cell cycle analysis (Figure 5C). Along with cell death, immunoblotting demonstrated that ON 01910.Na induced hyperphosphorylation of RanGAP1, increased expression of RanGAP1.SUMO1 but decreased expression of free unmodified RanGAP1 (Figure 5D). Interestingly, ON 01910.Na showed no cytotoxicity for normal CD3^+^ T cells and CD19^+^ B cells (Figure S3 in File S1).

## Discussion

Using proteomic analysis to compare DLCBL cell lines with LCL cells, we found the differential expression of RanGAP1, a cell-cycle regulator between DLBCL and reactive lymphoid hyperplasia. As a cell-cycle regulator, RanGAP1 was also frequently overexpressed in other BCL with brisk mitotic activity (lymphoblastic lymphoma/leukemia, and Burkitt lymphoma) or cell-cycle deregulation (mantle cell lymphoma and Hodgkin’s lymphoma) [36,37], but only occasionally in low-grade BCL. Interestingly, serum levels of RanGAP1 were higher in patients with high-grade BCL than in low-grade BCL and healthy controls. *In vitro*, RNA interference with RanGAP1 showed no effects on non-neoplastic LCL cells but induced DLBCL cell death and cell-cycle arrest, by inhibiting the expression of Aurora kinases and TPX2, the crucial regulators of mitosis and cytokinesis. Interestingly, ON 01910.Na, a styryl benzylsulfone capable of multikinase inhibition was selectively cytotoxic for DLBCL. Our findings suggest that RanGAP1 is a promising therapeutic target for DLBCL.

Ran is a nuclear Ras-like GTPase involved in nuclear transport, RNA processing, cell-cycle progression, and mitotic spindle formation [32]. The nuclear import cycle is orchestrated by the GTP- and GDP-bound states of Ran, which is regulated by nuclear guanine nucleotide-*e*xchange *f*actor (RanGEF, also known as RCC1, *r*egulator of *c*hromosome *c*ondensation 1) and cytoplasmic RanGAP1 [38,39]. During mitosis, Ran is involved in mitotic spindle assembly, and RanGAP1 is associated with mitotic spindles that are particularly concentrated near kinetochores [32]. RanGAP1 conjugation with SUMO-1 is required for mitotic localization and is important for spatially regulating the Ran pathway during mitosis [32,40]. Taking all these findings together, RanGAP1 appears to be a key regulator of the Ran GTP/GDP cycle and involved in cell-cycle control.

The *RANGAP1* knock-out mice were embryonically lethal, which highlights the pivotal function of the *RANGAP1* gene in cell survival [41]. Animal models with a conditional knockout of RanGAP1 in B cells are needed to clarify its function in B-cell development during different stages. Because RanGAP1 is expressed in germinal centers of lymph nodes and BCL with a high-growth fraction, it seems likely to be involved in cell-cycle progression [42]. Indeed, we demonstrated that inhibiting RanGAP1 expression increased DLBCL cell death and cell-cycle arrest. Moreover, RanGAP1-specific siRNAs also inhibited the expression of Aurora kinases and TPX2, the key regulators of mitotic cell division, and clinical indicators of aggressive cancers. We thus suggest that downregulation of RanGAP1 induces DLBCL cell-cycle arrest and death by inhibiting the expression of Aurora kinases and TPX2. TPX2, *t*argeting *p*rotein for XKLP2 (*Xenopus* kinesin-*l*ike *p*rotein 2), is a multifaceted protein for mitosis, including microtubule nucleation and targeting Aurora-A to the spindle [35]. TPX2-induced activation of Aurora-A is essential for Ran-stimulated spindle assembly [43]. Aurora kinases, a novel family of serine/threonine kinases, are substantially involved in mitotic cell division, overexpressed in many human cancers, and correlated with chromosomal instability and clinically aggressive disease [28,34]. The signals for mitotic spindle assembly contain at least two parts: one is the RanGTP signal where Aurora-A acts downstream; the other is the Aurora-B signal generated by localization of Aurora-B kinase [35,44]. Our finding that RanGAP1 knockdown inhibited the expression of Aurora-A and -B suggests that RanGAP1 may be more important than previously thought. Therefore, RanGAP1 is not merely a marker of cell division, because it is also highly expressed in mantle cell and Hodgkin’s lymphomas, both of which have relatively lower proliferation activity (Figure S4 in File S1). Aurora kinases are expressed and active at the highest level during the G2/M phase of the cell cycle [34]. We also found that RanGAP1 knockdown downregulated the expression of Aurora kinases that was correlated with cell-cycle arrest in the G0/G1 phase in DLBCL cells. In contrast, the cytotoxic effect of ON 01910.Na was through prolonged phosphorylation/hyperphosphorylation of RanGAP1.SUMO1 followed by M-phase arrest and the consequent induction of cell death [45]. 

Many articles have addressed aspects of the molecular biology of RanGAP1, such as the interacting molecules and the regulatory mechanisms [31,32,38]. However, its role in reactive and neoplastic B lymphocytes has not been addressed. In the literature, there is only one article showing RanGAP1 expression in LBCL cell lines [46]. In general, BCL can be divided into low- and high-proliferation fraction categories. The primary pathogenesis of the former depends on inhibiting apoptosis, such as the overexpression of BCL2 and API2, whereas the latter is characterized by brisk proliferation through the dysfunction of cell-cycle regulators [17,37]. Here, we demonstrated that RanGAP1 was highly expressed in BCL with brisk mitotic activity or cell-cycle deregulation [36,37]. Furthermore, inhibiting RanGAP1 expression increased DLBCL tumor cell death and cell-cycle arrest but showed no effect on LCL cells. The selective overexpression of RanGAP1 in aggressive B-cell and Hodgkin’s lymphomas may shed light on innovative targeted therapy. Oussenko et al [45] recently reported that ON 01910.Na, an inhibitor of RanGAP1, prolongs the hyperphosphorylation of RanGAP1, which consequently induces apoptosis rather than direct DNA damage. We found a similar effect of ON 01910.Na on DLBCL cells. Furthermore, ON 01910.Na showed absent or only minimal cytotoxicity for normal B and T cells (Figure S3 in File S1). Given that ON 01910.Na is currently under a randomized phase III trial for patients with refractory myelodysplastic syndrome [47], this drug would be very promising for the RanGAP1-targeted lymphoma therapy.

Proteomic analysis that compares tumorous and non-tumorous cells is a powerful tool for discovering tumor-specific proteins [48]. By comparing whole lysates of tumorous cells with those of non-tumorous cells, we found that RanGAP1 was differentially expressed in reactive and neoplastic B-cell proliferations, as well as in BCL with low- and high-proliferation fractions. Interestingly, the serum level of RanGAP1 in patients with high-grade BCL was higher than in low-grade BCL and healthy controls. Because RanGAP1 is present in cytoplasm and perinucleus, and no secreted form is found [49], it is likely that the serum level arises from the death of tumor cells. Thus, the higher serum level might represent more tumor cell death at diagnosis, and might have no prognostic significance and show no correlation with other clinicopathologic factors [50,51]. Although serum RanGAP1 level was significantly higher in patients with high-grade BCL, its poor sensitivity may limit the clinical utility.

In conclusion, using comparative proteomic analysis, we found that RanGAP1, a cell-cycle coordinator, was present in the tumor tissues and patient serum of high-grade BCL. *In vitro*, by inhibiting Aurora kinases and TPX2, knockdown of RanGAP1 increased tumor cell death and cell-cycle arrest but had no effect on non-neoplastic cells. Besides, ON 01910.Na induced hyperphosphorylation of RanGAP1.SUMO1, mitotic cell arrest and consequent cell death. Therefore, RanGAP1 is an appropriate lymphoma marker with the potential for tumor-targeted therapy [45].

## Supporting Information

File S1 **Supporting Materials and Methods.** Culturing DLBCL and B-lymphoblastoid cell lines. Proteomic analysis. Enzyme-linked immunosorbent assay (ELISA) for RanGAP1. Transfecting RANGAP1-specific shRNA into cell lines. Quantitative real-time polymerase chain reaction (Q-PCR). Figure S1. The standard curve of RanGAP1 serum level. Figure S2. Higher serum level of RanGAP1 in patients with high-grade BCL than in low-grade BCL and healthy controls. Figure S3. No cytotoxic effect on B or T cells from healthy donors at 48 hours. Figure S4. Double staining of RanGAP1 and Ki-67 in mantle cell and Hodgkin lymphomas. Table S1. Protein identification data expressed according to Paris guidelines.
(PDF)
