# Supplementary material for: Ran GTPase-Activating Protein 1 Is a Therapeutic Target in Diffuse Large B-Cell Lymphoma
Source: PLoS One. 2013 Nov 6;8(11):e79863. doi: 10.1371/journal.pone.0079863 (PMC3819250; doi:10.1371/journal.pone.0079863)
Supplement: File S1 — Supporting Materials and Methods. Culturing DLBCL and B-lymphoblastoid cell lines. Proteomic analysis. Enzyme-linked immunosorbent assay (ELISA) for RanGAP1. Transfecting RANGAP1-specific shRNA into cell lines. Quantitative real-time polymerase chain reaction (Q-PCR). Figure S1. The standard curve of RanGAP1 serum level. Figure S2. Higher serum level of RanGAP1 in patients with high-grade BCL than in low-grade BCL and healthy controls. Figure S3. No cytotoxic effect on B or T cells from healthy donors at 48 hours. Figure S4. Double staining of RanGAP1 and Ki-67 in mantle cell and Hodgkin lymphomas. Table S1. Protein identification data expressed according to Paris guidelines. (PDF) [file pone.0079863.s001.pdf]

Supporting Information File S1 for

Ran GTPase-activating protein 1 is a therapeutic target in diffuse large B-cell lymphoma

Kung-Chao Chang[1*], Wei-Chao Chang[2†], Yao Chang[3], Liang-Yi Hung[4], Chien-Hsien Lai[4], Yu-Min Yeh[5], Yu-Wei Chou[1] and Chung-Hsuan Chen[2*]

[1]Department of Pathology, and [5]Department of Internal Medicine, National Cheng Kung University and Hospital, Tainan, Taiwan;

[2]Genomics Research Center, Academia Sinica, Taipei, Taiwan;

[3]Division of Infectious Diseases, National Health Research Institute, Tainan, Taiwan;

[4]Institute of Bioinformatics and Biosignal Transduction, National Cheng Kung University, Tainan, Taiwan

[†]New affiliation information: The Graduate Institute of Cancer Biology and Center for Molecular Medicine, China Medical University and Hospital, Taichung, Taiwan

***Correspondence:**

Kung-Chao Chang, MD, PhD, Department of Pathology, National Cheng Kung University Hospital, 138 Sheng-Li Road, Tainan, 704 Taiwan, Tel: 886-6-235-3535 ext. 2636; Fax: 886-6-276-6195; E-mail: changkc@mail.ncku.edu.tw or

Chung-Hsuan Chen, PhD, The Genomics Research Center, Academia Sinica, 128 Academia Road, Sec. 2, Nankang, Taipei 115, Taiwan, Tel: 886-2-2787-1200; Fax: 886-2-2789-9923; E-mail: winschen@gate.sinica.edu.tw

Supporting Materials and Methods

Culture of DLBCL and B-lymphoblastoid cell lines

The cells (HT, SU-DHL-5 and EBV-transformed B-lymphoblastoid cell line [LCL]) were cultured at 37°C and 7% CO_2 in RPMI 1640 medium (Gibco/BRL, Grand Island, NY, USA) supplemented with 10% heat-inactivated FCS, 4 mM of glutamine, 75 units/ml of streptomycin, and 100 units/ml of penicillin. Before the analysis, the DLBCL and LCL lines were washed three times with PBS to minimize contamination from serum. Cell viability was determined using the trypan blue exclusion test. Samples from whole cell lysate were normalized based on protein concentration and labeled for comparative proteomics analysis.

Proteomic analysis

Protein separation and in-gel digestion

Each 5×10^5 cells were lysed by boiling and each 30 µg of extracted protein was applied to 10% polyacrylamide gel electrophoresis. The gel was subsequently visualized through Coomassie blue-staining. Gel lanes were divided into 18 sections and all slices were cut into small gel pieces (< 1 mm^3), followed by in-gel digestion. All in-gel digestions of proteins were done manually in a laminar flow hood to reduce the amount of keratin contamination. The gel pieces were destained with 50% acetonitrile (ACN) and 25 mM of ammonium bicarbonate. Then, the gel pieces were placed in 10 mM of dithiothreitol for 45 min at 56°C to reduce disulfide bonds. An alkylation reaction was performed using freshly prepared iodoacetamide (55 mM) for 45 min at room temperature in the dark. Sequencing grade trypsin (final concentration: 4 ng/µl) was used to digest protein at 37°C for 16-18 h. Tryptic peptides were extracted using a 60% ACN/1% trifluoroacetic acid solution. After the peptides had been dried to remove the solvent, the re-dissolved tryptic peptides were subjected to

LC LTQ-FT ICR MS analysis.

LC LTQ-FT ICR MS analysis

MS/MS experiments were performed with an LTQ-FT ICR MS (Thermo Electron, San Jose, CA, USA) equipped with a nano-electrospray ion source (New Objective, Woburn, MA, USA), a binary HPLC pump (1100 Series; Agilent Technologies, Santa Clara, CA, USA) and an autosampler (Famos 900 / 920 LC Packings, Sunnyvale, CA, USA). Tryptic peptide mixtures were injected at a 10 μL/min flow rate into a self-packed pre-column in line with a reverse phase C_{18} nano-column (75 μm I.D. × 200 mm) that used resin (particle size, 5 μm; pore size, 200 Å) (Magic C_{18}AQ; Michrom Bioresources, Auburn, CA, USA). The analytic program was set at a linear gradient from 5% to 50% ACN with a 60-min running cycle and a split flow rate of 300 nl/min. The full-scan survey MS experiment (*m*/*z* 320-2,000) was executed in FT ICR MS with a mass resolution of 100,000 at *m*/*z* 400. The top ten most abundant multiply charged ions were sequentially isolated for MS/MS by LTQ. Singly charged ions were rejected for MS/MS sequencing.

Mascot search and label-free quantitative analysis

The *.raw files of spectra were converted to *.mgf files (Mascot Daemon 2.2; Matrix Science Ltd., London, UK) and merged into a single file for searching using Mascot Daemon software based on the IPI human database (v 3.66). The following Mascot Daemon parameter settings were used: the peptide tolerance was 15 ppm with 2^+ and 3^+ peptide charges and the MS/MS tolerance was 0.6 Da. Two missed cleavages by trypsin were allowed; carbamidomethyl (C) was used as a fixed modification; and oxidation (M) and deamidated (NQ) were used as variable modifications. The significance threshold for the identification was set as $p < 0.01$, and at least two peptides were matched. Label-free quantitative analysis was done

using quantitative proteomics software (MaxQuant; Max Planck Institute of Biochemistry, Munich, Germany. Available at: http://www.maxquant.org/) [22].

Enzyme-linked immunosorbent assay (ELISA) for RanGAP1

Polystyrene 96 well ELISA plates (eBioscience, San Diego, CA, USA) were incubated overnight at 4°C with 100 μl of coating buffer (0.1 M $NaHCO_3$ [pH = 9.6]) containing 0.2 μg/ml of purified capture monoclonal antibody (mAb, RanGAP1, C-5; Santa Cruz). The wells were blocked with 100 μl of PBS with 10% FBS for 1 h at room temperature (RT), and triplicates of 100 μl of serially diluted standard (RanGAP1 full-length recombinant protein, Novus Biologicals, Littleton, CO, USA) or test samples at various dilutions were added and incubated for 2 h at 37°C. Control and samples as well as subsequent detection reagents were diluted in PBS with 0.05% Tween 20. The fluid from the wells was aspirated and the plates were washed and then incubated for 1 h at RT with 100 μl of detection polyclonal Ab (RanGAP1, N-19, sc-1862; Santa Cruz) at 0.8 μg/ml. Subsequently, the wells were washed and then incubated with 100 μl of goat IgG-HRP conjugate (1:2500, Dako) for 1 h at RT. Between incubations, the plates were washed with PBS containing 0.05% Tween 20. Finally, 100 μl of tetramethylbenzidine (TMB, ES001-500ML; Millipore, Billerica, MA, USA) buffer was added to each well and incubated in the dark for 30 min. After adding stop solution (2N H_2SO_4), the optical density (OD) was measured with a spectrophotometer (μQuant, BIO-TEK instruments, Inc., Winooski, VT, USA) at 450 nm.

Transfecting RANGAP1-specific shRNA into cell lines

The sequences for the RANGAP1 shRNA were as follows:

5′-GATCCCCCAAGAGTGAAGACAAGGTCAATTCAAGAGATTGACCTTGTCT TCACTCTTGTTTTTGGAAA-3′ (forward), and

5′-AGCTTTTCCAAAAACAAGAGTGAAGACAAGGTCAATCTCTTGAATTGACCTTGTCTTCACTCTTGGGG-3′ (reverse). shRNAs were controlled for sequence specificity using a BLAST search and did not show homology to other known human genes. All shRNAs were purchased from Purigo Biotech (Taipei, Taiwan). The plasmids expressing gene-specific shRNA were constructed using synthetic oligonucleotides cloned into the *Bgl*II/*Hind*III cloning sites of the pSUPER-EGFP vector (pSUPER RNAi System; OligoEngine, Seattle, WA, USA). Each of the shRNA expression plasmids had a 21 nucleotide gene-specific target in both sense and antisense orientation, separated by a 9 nucleotide spacer sequence. The 5′ end corresponded to the *Bgl*II site, while the 3′ end contained the five-thymidine sequence and *Hind*III-corresponding nucleotides. In all siRNA (small interfering RNA) experiments, a control sample treated with empty vector was included. Other two sets of siRNA sequences were as follows: siRNA1, 5’-GATCCCCGAACTTGTCATTCTGTGAAATTTCAAGAGAATTTCACAGAATGACAAGTTCTTTTTGGAAA-3’ (forward), and 5’-AGCTTTTCCAAAAAGAACTTGTCATTCTGTGAAATTCTCTTGAAATTTCACAGAATGACAAGTTCGGG-3’ (reverse); siRNA2, 5’-GATCCCCCTGCCTTCCTAAAGGTGTCATTTCAAGAGAATGACACCTTTAGGAAGGCAGTTTTTGGAAA-3’ (forward), and 5’-AGCTTTTCCAAAAACTGCCTTCCTAAAGGTGTCATTCTCTTGAAATGACACCTTTAGGAAGGCAG GGG-3’ (reverse).

An shRANGAP1 expression construct was transfected using an electroporation machine (Microporator; Digital Bio Technology, Suwon, Korea) with 6-8 μg of DNA at 1100-1300 V for 20-30 ms [23]. Transfection efficiency was determined by flow cytometric analysis on fluorescent cells with a flow cytometer (FACSCalibur with

CellQuest Pro 4.0.2; Becton Dickinson, Franklin Lakes, NJ, USA) [27].

Quantitative real-time polymerase chain reaction (Q-PCR)

Primers used for Q-PCR were described as followed: *Aurora-A* forward, 5´-AATGCCCTGTCTTACTGTCATTC-3´ and reverse, 5´-TCCAGAGATCCACCTTCTCATC-3´; *Aurora-B* forward, 5’-CATGGAGGAGTTGGCAGATGC-3’ and reverse 5’-TTAGGTCCACCTTGACGATGC-3’; *actin* forward, 5’-CTGGACTTCGAGCAAGAGATG-3’ and reverse 5’-TGATGGAGTTGAAGGTAGTTTCG-3’.

Supporting Figure S1

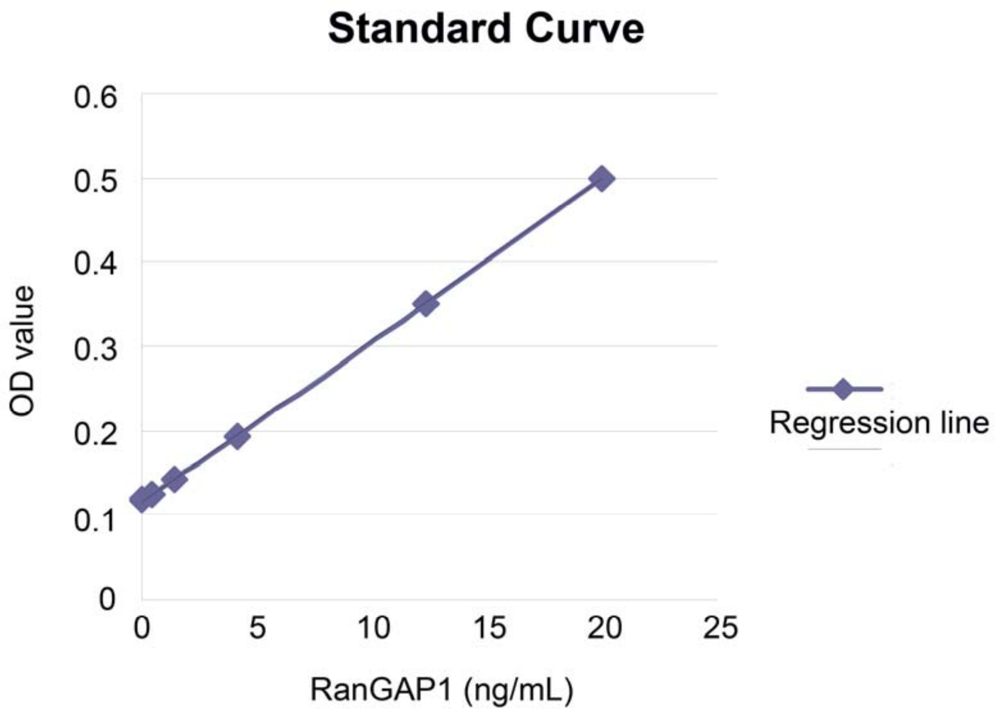

Supporting Figure S1. The standard curve of RanGAP1 serum level.

The colorimetric noncompetitive (immunometric sandwich assay) ELISA has been developed and the standard curve was established by testing triplicates of serially diluted standard. The detection range was 0-20 ng/mL (OD: 0.11-0.5). The regression line formula is: $y=0.0191x+0.116$, $R^2=0.9998$.

Supporting Figure S2.

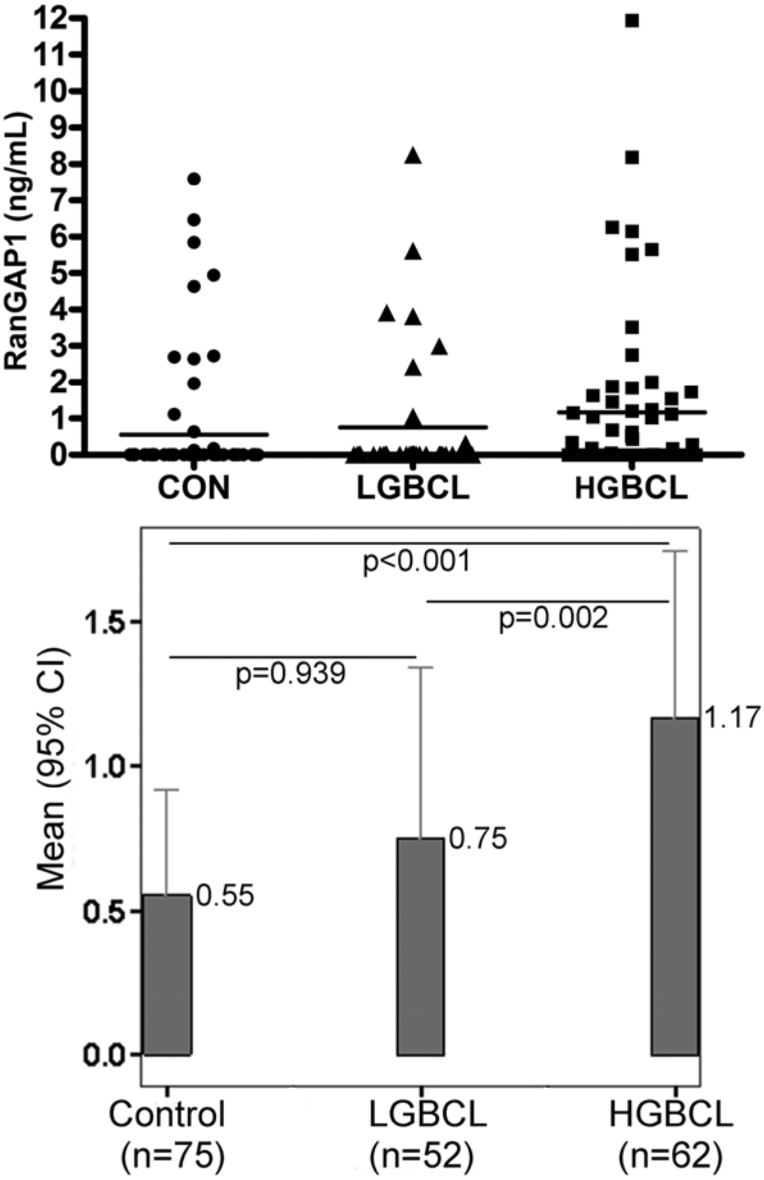

Supporting Figure S2. Higher serum level of RanGAP1 in patients with high-grade BCL than in low-grade BCL and healthy controls. Serum levels of RanGAP1 were higher in patients with high-grade BCL (1.71 ± 2.28 ng/mL, n = 62) than in low-grade BCL (0.75 ± 2.12 ng/mL, n = 52) and healthy controls (0.55 ± 1.58 ng/mL, n = 75) (high-grade BCL vs. low-grade BCL, p = 0.002; high-grade BCL vs. control, p < 0.001, Mann-Whitney U test). However, the RanGAP1 serum level was not so sensitive since half (n = 31) cases of high-grade BCL were not elevated.

Supporting Figure S3

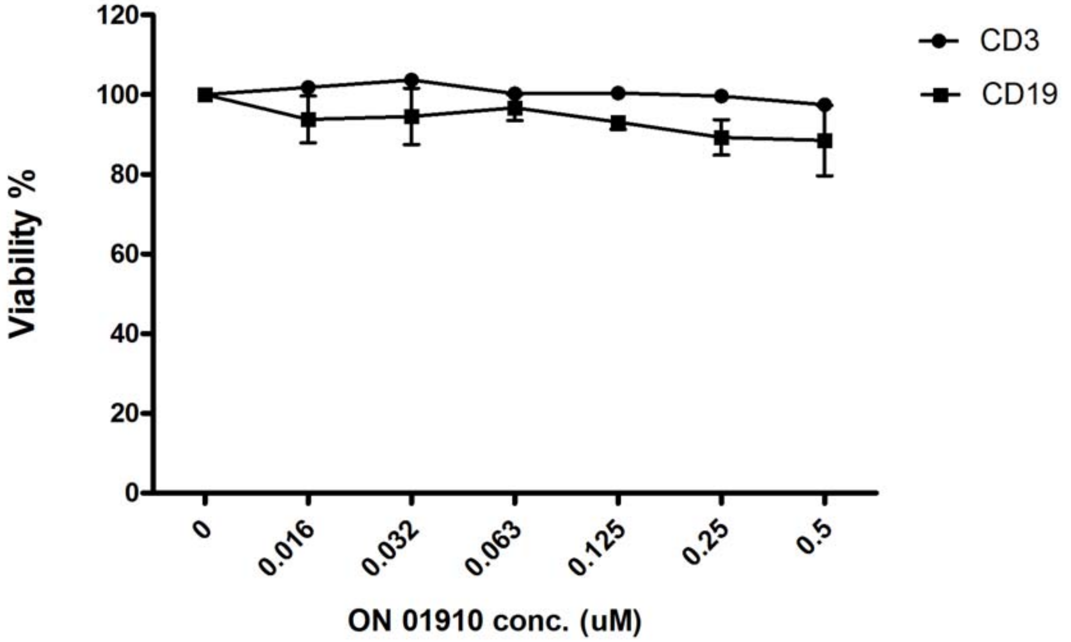

Supporting Figure S3. No cytotoxic effect on B or T cells from healthy donors at 48 hours. Peripheral blood mononuclear cells of healthy donors were treated with ON 01910.Na at increasing doses for 48 hours. Then, cell viability was assessed by flow cytometry by gating $CD3^+$ T cells and $CD19^+$ B cells. The percentage of viable cells normalized to the untreated control is shown.

Supporting Figure S4

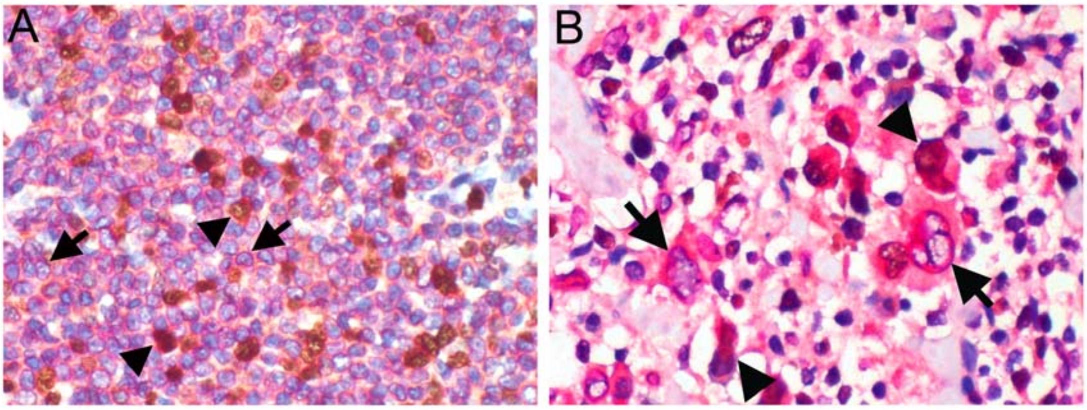

Supporting Figure S4. Double staining of RanGAP1 (red in cytoplasm) and Ki-67 (a proliferation marker, brown in nuclei) in mantle cell lymphoma (A) and Hodgkin lymphoma (B). Many RanGAP1-positive tumor cells were negative for Ki-67 staining in mantle cell (A, ×400) and Hodgkin lymphomas (B, ×400) (arrow: RanGAP1^{+}/Ki-67^{-}; arrowhead: RanGAP1^{+}/Ki-67^{+}).

Supporting Table S1. Protein identification data expressed according to Paris guidelines

Uniprot Acc#	Protein Name	m/z	z	Error (ppm)	Sequence	Score
P23526	Adenosylhomocysteinase	780.3815	2	-0.5853	ALDIAENEMPGLMR	93
P23526	Adenosylhomocysteinase	550.2774	3	0.1819	GISEETTTGVHNLYK	100
P23526	Adenosylhomocysteinase	944.9854	4	-1.1118	GISEETTTGVHNLYKMMANGILKVPAINVNDSVTK	41
P23526	Adenosylhomocysteinase	573.9517	3	-0.5940	KALDIAENEMPGLMR	119
P23526	Adenosylhomocysteinase	460.9209	3	-0.2246	KLDEAVAEAHLGK	137
P23526	Adenosylhomocysteinase	612.3510	3	-0.1584	KLDEAVAEAHLGKLNVK	74
P23526	Adenosylhomocysteinase	705.7149	3	1.2895	MMANGILKVPAINVNDSVTK	100
P23526	Adenosylhomocysteinase	668.0301	3	-0.1086	SDKLPYKVADIGLAAWGR	123
P23526	Adenosylhomocysteinase	420.5328	3	0.1559	SKFDNLYGCR	60
P23526	Adenosylhomocysteinase	419.5752	3	-0.1964	VADIGLAAWGRK	107
P23526	Adenosylhomocysteinase	631.0000	3	-0.2310	VAVVAGYGDVGKGCAQALR	97
P23526	Adenosylhomocysteinase	793.7499	3	0.6960	VAVVAGYGDVGKGCAQALRGFGAR	59
P23526	Adenosylhomocysteinase	356.8769	3	-0.0880	VNIKPQVDR	85
P23526	Adenosylhomocysteinase	463.2650	3	-0.2040	VNIKPQVDRYR	60
P23526	Adenosylhomocysteinase	393.2278	3	-0.0462	YSASKPLKGAR	81
P27707	Deoxycytidine kinase	450.9066	3	-0.3478	DAEKPVLFFER	110
P27707	Deoxycytidine kinase	481.2961	3	-0.2589	IKKISIEGNIAAGK	74
P27707	Deoxycytidine kinase	531.2996	3	-0.0249	LKDAEKPVLFFER	109
P27707	Deoxycytidine kinase	686.7935	2	0.2152	SCPSFSASSEGTR	64
P27707	Deoxycytidine kinase	792.6755	3	0.8743	WCNVQSTQDEFEELTMSQK	178

P52701	DNA mismatch repair protein Msh6	881.4405	3	-0.0823	AAAAPGASPSPGGDAAWSEAGPGPRPLAR	78
P52701	DNA mismatch repair protein Msh6	889.7889	3	0.8626	GGDGPMCRPVILLPEDTPPFLELK	58
P52701	DNA mismatch repair protein Msh6	369.4505	4	-0.4559	GGHFYSAKPEILR	51
P52701	DNA mismatch repair protein Msh6	401.7283	4	0.1167	LANLPEEVIQKGHR	108
P52701	DNA mismatch repair protein Msh6	433.7521	4	-0.1229	LANLPEEVIQKGHRK	61
P52701	DNA mismatch repair protein Msh6	336.2026	3	0.4997	LLKPYTGSK	103
P52701	DNA mismatch repair protein Msh6	594.3041	3	-0.6350	NRYQLEIPENFTTR	71
P52701	DNA mismatch repair protein Msh6	512.6056	3	0.2851	QATSISSETKNTLR	97
P52701	DNA mismatch repair protein Msh6	429.8932	3	0.0529	SPALSDANKASAR	88
P52701	DNA mismatch repair protein Msh6	923.1288	3	-0.0671	SSLSCSLQEGLIPGSQFWDASKTLR	47
P52701	DNA mismatch repair protein Msh6	660.7174	3	0.0670	STVDAEAVHKLLTLIKEL	61
P52701	DNA mismatch repair protein Msh6	617.3067	4	0.3812	TLFSTHYHSLVEDYSQNVAVR	118
P52701	DNA mismatch repair protein Msh6	486.2488	3	0.2446	TLLEEEYFREK	101
P49736	DNA replication licensing factor MCM2	633.9549	3	-0.1862	AESSESFTMASSPAQRR	70
P49736	DNA replication licensing factor MCM2	1021.4823	3	-0.1481	AIPELDAYEAEGLALDDEDVEELTASQR	63
P49736	DNA replication licensing factor MCM2	773.3478	4	0.4157	ATEDGEEDEEMIESIENLEDLKGHSVR	69
P49736	DNA replication licensing factor MCM2	883.1533	3	-0.1071	DNNELLLFILKQLVAEQVTYQR	123
P49736	DNA replication licensing factor MCM2	362.8636	3	-0.0792	DREAGRGLGR	60
P49736	DNA replication licensing factor MCM2	744.8536	2	0.7520	DTVDPVQDEMLAR	120
P49736	DNA replication licensing factor MCM2	831.0822	3	0.4679	FDILCVVRDTVDPVQDEMLAR	54
P49736	DNA replication licensing factor MCM2	678.0126	3	-0.6861	FGAQQDTIEVPEKDLVDK	43
P49736	DNA replication licensing factor MCM2	565.5459	4	-0.1379	FGAQQDTIEVPEKDLVDKAR	124
P49736	DNA replication licensing factor MCM2	586.8373	2	0.5195	GLALALFGGEPK	97

P49736	DNA replication licensing factor MCM2	542.6385	3	0.1122	GLALALFGGEPKNPGGK	96
P49736	DNA replication licensing factor MCM2	631.9536	3	0.1116	GLLYDSDEEDEERPAR	46
P49736	DNA replication licensing factor MCM2	480.0112	4	-0.1332	IFASIAPSIYGHEDIKR	74
P49736	DNA replication licensing factor MCM2	522.7690	4	-0.6374	IHLRDYVIEDDVNMAIR	61
P49736	DNA replication licensing factor MCM2	343.2013	3	0.0162	IRIQESPGK	124
P49736	DNA replication licensing factor MCM2	435.9224	3	-0.1752	ISHLPLVEELR	100
P49736	DNA replication licensing factor MCM2	556.3310	5	-0.3008	ISHLPLVEELRSLRQLHLNQLIR	50
P49736	DNA replication licensing factor MCM2	330.5262	3	-0.0166	ITNHIHVR	56
P49736	DNA replication licensing factor MCM2	435.8871	3	-0.1826	LNQMDQDKVAK	81
P49736	DNA replication licensing factor MCM2	576.2973	4	-0.5071	NRFGAQQDTIEVPEKDLVDK	51
P49736	DNA replication licensing factor MCM2	378.8963	3	-0.3042	QLHLNQLIR	99
P49736	DNA replication licensing factor MCM2	497.6353	3	0.5051	SLRQLHLNQLIR	103
P49736	DNA replication licensing factor MCM2	554.9414	3	-0.0827	THVDSHGHNVFKER	92
P49736	DNA replication licensing factor MCM2	660.6762	3	-1.7177	TSGVVTSCTGVLPQLSMVK	69
P49736	DNA replication licensing factor MCM2	327.8503	3	-0.0673	YIEKVSSR	52
P25205	DNA replication licensing factor MCM3	878.1106	3	-0.6099	AIPTTGRGSSGVGLTAAVTTDQETGER	120
P25205	DNA replication licensing factor MCM3	538.2909	3	0.0764	ALKDFVASIDATYAK	83
P25205	DNA replication licensing factor MCM3	669.0253	3	-0.1994	ANRLLNNAFEELVAFQR	110
P25205	DNA replication licensing factor MCM3	692.3387	3	0.2754	CSVLAAANPVYGRYDQYK	54
P25205	DNA replication licensing factor MCM3	771.0775	3	-0.1079	IIKPVLTQESATYIAEEYSR	101
P25205	DNA replication licensing factor MCM3	645.8563	4	-0.0550	IIKPVLTQESATYIAEEYSRLR	51
P25205	DNA replication licensing factor MCM3	404.5788	3	0.2523	LIVNVNDLRR	87
P25205	DNA replication licensing factor MCM3	555.2983	3	-0.4969	LLNNAFEELVAFQR	161

P25205	DNA replication licensing factor MCM3	485.2282	3	0.4832	LRSQDSMSSDTAR	62
P25205	DNA replication licensing factor MCM3	388.8802	3	0.1901	SKDIFDQLAK	118
P25205	DNA replication licensing factor MCM3	397.5377	3	-0.5910	SVHYCPATKK	54
P25205	DNA replication licensing factor MCM3	718.0170	3	0.3516	TADSQETKESQKVELSESR	65
P25205	DNA replication licensing factor MCM3	513.3069	3	-0.4061	TLETLIRLATAHAK	106
P25205	DNA replication licensing factor MCM3	777.7179	3	-0.0114	TVDLQDAEEAVELVQYAYFK	171
P25205	DNA replication licensing factor MCM3	820.4162	3	0.2187	TVDLQDAEEAVELVQYAYFKK	99
P25205	DNA replication licensing factor MCM3	464.9125	3	0.3093	VRELISDNQYR	64
P33991	DNA replication licensing factor MCM4	786.3825	3	0.3919	DMFEEALRALADDDFLTVTGK	61
P33991	DNA replication licensing factor MCM4	469.2527	3	-0.2505	ELSRKPDIYER	80
P33991	DNA replication licensing factor MCM4	940.8090	3	-0.5629	FIDPLAKEEENVGIDITEPLYMQR	136
P33991	DNA replication licensing factor MCM4	877.1032	3	-1.1575	GLQVDLQSDGAAAEDIVASEQSLGQK	94
P33991	DNA replication licensing factor MCM4	633.3157	3	-0.3575	GSSAVGLTAYVMKDPETR	55
P33991	DNA replication licensing factor MCM4	765.7316	3	-0.5290	LGEINVIGEPFLNVNCEHIK	127
P33991	DNA replication licensing factor MCM4	468.9889	4	0.2407	LHGLDEEAEQKLFSEK	78
P33991	DNA replication licensing factor MCM4	779.7297	6	0.2085	LQESPEDMPAGQTPHTVILFAHNDLVDKVQPGDRVNVTGIYR	31
P33991	DNA replication licensing factor MCM4	690.3455	3	-0.1658	LSEEASQALIEAYVDMRK	109
P33991	DNA replication licensing factor MCM4	567.6441	3	0.1485	LSNKVEAIDVEEAKR	152
P33991	DNA replication licensing factor MCM4	714.3805	3	0.0792	NLNPEDIDQLITISGMVIR	252
P33991	DNA replication licensing factor MCM4	367.2000	3	-0.0429	QRPDLGSAQK	124
P33991	DNA replication licensing factor MCM4	508.0142	4	-0.2248	RLHGLDEEAEQKLFSEK	81
P33991	DNA replication licensing factor MCM4	414.9225	3	0.3475	RVELLKELSR	71
P33991	DNA replication licensing factor MCM4	348.1821	3	0.0499	SFDKNLYR	106

P33991	DNA replication licensing factor MCM4	408.4730	4	0.1329	SVYKTHIDVIHYR	75
P33991	DNA replication licensing factor MCM4	440.4967	4	-0.0311	SVYKTHIDVIHYRK	58
P33991	DNA replication licensing factor MCM4	911.9826	2	1.4597	TGIVDISILTTGMSATSR	54
P33991	DNA replication licensing factor MCM4	652.0179	3	-0.7997	TSVLAAANPIESQWNPKK	101
P33991	DNA replication licensing factor MCM4	461.2613	2	-0.1477	VNVTGIYR	76
P33992	DNA replication licensing factor MCM5	582.8133	2	1.0803	AIACLLFGGSR	88
P33992	DNA replication licensing factor MCM5	595.2787	3	-0.9109	CPLDPYFIMPDKCK	102
P33992	DNA replication licensing factor MCM5	714.3708	4	0.3975	EVADEVTRPRPSGEEVLQDIQVMLK	84
P33992	DNA replication licensing factor MCM5	502.5191	4	-0.6157	FAIGSQVSEHSIIKDFTK	52
P33992	DNA replication licensing factor MCM5	589.2881	3	0.2412	GSSAAGLTASVMRDPSSR	83
P33992	DNA replication licensing factor MCM5	568.9506	3	-0.1496	LQELPDAVPHGEMPR	94
P33992	DNA replication licensing factor MCM5	696.3737	3	0.1428	NTLTNIAMRPGLEGYALPR	84
P33992	DNA replication licensing factor MCM5	332.5310	3	-0.3416	RFKEFLR	92
P33992	DNA replication licensing factor MCM5	562.9663	3	1.0444	RGDINLLMLGDPGTAK	124
P33992	DNA replication licensing factor MCM5	613.6271	3	0.0926	SFAGAVSPQEEEEFRR	125
P33992	DNA replication licensing factor MCM5	917.7196	3	0.3605	SGFDDPGIFYSDSFGGDAQADEGQAR	67
P33992	DNA replication licensing factor MCM5	484.5903	3	0.4230	SIAPSIFGGTDMKK	75
Q14566	DNA replication licensing factor MCM6	334.2015	3	0.5455	AGVKATLNAR	64
Q14566	DNA replication licensing factor MCM6	687.8664	4	-0.6408	ALGVRDLSYRLVFLACCVAPTNPR	85
Q14566	DNA replication licensing factor MCM6	708.3772	3	-0.1762	EIPLAKDFYVAFQDLPTR	132
Q14566	DNA replication licensing factor MCM6	598.3003	3	0.7555	ESEDFIVEQYKHLR	49
Q14566	DNA replication licensing factor MCM6	365.2049	3	0.2775	FLLDTNKSR	96
Q14566	DNA replication licensing factor MCM6	346.8626	3	-0.2413	FVDFQKVR	112

Q14566	DNA replication licensing factor MCM6	735.3989	3	4.5709	FVDFQKVRIQETQAELPR	98
Q14566	DNA replication licensing factor MCM6	339.4543	4	0.0920	HVKEAFRLLNK	54
Q14566	DNA replication licensing factor MCM6	565.6443	3	0.3425	IQETQAELPRGSIPR	74
Q14566	DNA replication licensing factor MCM6	531.0298	4	0.6264	ISKESEDFIVEQYKHLR	69
Q14566	DNA replication licensing factor MCM6	521.2617	3	1.3634	KVEEEEDESALKR	164
Q14566	DNA replication licensing factor MCM6	884.7572	3	-0.5284	MDLAAAAEPGAGSQHLEVRDEVAEK	113
Q14566	DNA replication licensing factor MCM6	562.6250	3	0.3377	TSILAAANPISGHYDR	121
Q14566	DNA replication licensing factor MCM6	480.6037	3	0.1131	VRIQETQAELPR	119
Q14566	DNA replication licensing factor MCM6	569.9602	3	-0.2810	VSGVDGYETEGIRGLR	114
P33993	DNA replication licensing factor MCM7	450.5819	3	0.2258	ALKDYALEKEK	125
P33993	DNA replication licensing factor MCM7	662.8347	4	0.5299	EVVNKDVLDVYIEHRLMMEQR	79
P33993	DNA replication licensing factor MCM7	640.6790	3	0.5216	LFADAVQELLPQYKER	127
P33993	DNA replication licensing factor MCM7	418.2314	3	0.1677	QFKYGNQLVR	65
P33993	DNA replication licensing factor MCM7	609.3163	3	-0.0064	RFELYFQGPSSNKPR	126
P33993	DNA replication licensing factor MCM7	571.3099	4	-0.1619	YAKLFADAVQELLPQYKER	118
P33993	DNA replication licensing factor MCM7	382.2277	3	0.1045	ADSVGKLVTVR	96
P33993	DNA replication licensing factor MCM7	515.3062	2	0.3469	AGILTTLNAR	55
P33993	DNA replication licensing factor MCM7	517.6470	3	-0.3478	ALLLLLVGGVDQSPR	135
P33993	DNA replication licensing factor MCM7	420.8896	3	-0.1551	DSLLGDKGQTAR	84
P33993	DNA replication licensing factor MCM7	510.2984	3	0.0843	EVRADSVGKLVTVR	106
P33993	DNA replication licensing factor MCM7	698.3480	4	-0.8026	FIKFQEMKMQEHSDQVPVGNIPR	59
P33993	DNA replication licensing factor MCM7	456.8823	3	-0.2028	FSEAEQRCVSR	54
P33993	DNA replication licensing factor MCM7	995.1455	3	0.6224	GFTPAQFQAALDEYEELNVWQVNASR	131

P33993	DNA replication licensing factor MCM7	438.2698	3	0.0261	GIVTRVSEVKPK	63
P33993	DNA replication licensing factor MCM7	678.3896	3	-0.2999	IAQPGDHVSVTGIFLPILR	138
P33993	DNA replication licensing factor MCM7	499.7514	4	-0.1521	LMEMSKDSLLGDKGQTAR	67
P33993	DNA replication licensing factor MCM7	756.0133	3	-0.2404	MNKSEDDESGAGELTREELR	74
P33993	DNA replication licensing factor MCM7	569.6141	3	0.2587	MQEHSDQVPVGNIPR	50
P33993	DNA replication licensing factor MCM7	631.6205	3	-0.4835	SEDDESGAGELTREELR	92
P33993	DNA replication licensing factor MCM7	384.2158	3	0.0889	SGGRLYLQTR	138
P33993	DNA replication licensing factor MCM7	826.9701	2	0.0413	SLEQNIQLPAALLSR	110
P33993	DNA replication licensing factor MCM7	511.2912	3	-0.0429	SQLLSYIDRLAPR	101
P33993	DNA replication licensing factor MCM7	403.5388	3	-0.2140	SVRFSEAEQR	116
P33993	DNA replication licensing factor MCM7	768.7414	3	1.0386	TGFRQVVQGLLSETYLEAHR	118
P33993	DNA replication licensing factor MCM7	529.6972	5	-0.3452	TGFRQVVQGLLSETYLEAHRIVK	94
P33993	DNA replication licensing factor MCM7	491.9439	3	-0.2180	TQRPADVIFATVR	102
P33993	DNA replication licensing factor MCM7	543.8026	4	0.0384	TQRPADVIFATVRELVSGGR	107
C9J4C3	DNA topoisomerase 2	969.4456	3	1.0212	AGEMELKPFNGEDYTCITFQPDLSK	45
C9J4C3	DNA topoisomerase 2	423.2225	3	-0.0892	AYDIAGSTKDVK	80
C9J4C3	DNA topoisomerase 2	365.5380	3	-0.5069	DKYGVFPLR	65
C9J4C3	DNA topoisomerase 2	427.2434	3	-0.1949	DKYGVFPLRGK	112
C9J4C3	DNA topoisomerase 2	541.9494	3	0.2375	DPALNSGVSQKPDPAK	100
C9J4C3	DNA topoisomerase 2	468.5590	3	0.5509	EAKEYFADMKR	51
C9J4C3	DNA topoisomerase 2	543.6435	3	-1.0781	HRFLEEFITPIVK	151
C9J4C3	DNA topoisomerase 2	681.0491	3	-0.0277	HVDYVADQIVTKLVDVVK	162
C9J4C3	DNA topoisomerase 2	582.6442	3	-0.1266	IFDEILVNAADNKQR	123

C9J4C3	DNA topoisomerase 2	696.0358	3	-0.4381	IFDEILVNAADNKQRDPK	55
C9J4C3	DNA topoisomerase 2	389.5595	3	-0.0822	ILNVREASHK	55
C9J4C3	DNA topoisomerase 2	566.9810	3	-0.5322	IPNFDVREIVNNIR	72
C9J4C3	DNA topoisomerase 2	464.5129	4	-0.0750	IPNFDVREIVNNIRR	53
C9J4C3	DNA topoisomerase 2	469.5902	3	0.1319	ITIEMKAEAEKK	60
C9J4C3	DNA topoisomerase 2	425.5992	3	0.2726	KQTTLAFKPIK	84
C9J4C3	DNA topoisomerase 2	564.5063	4	-0.7549	KSKGESDDFHMDFDSAVAPR	52
C9J4C3	DNA topoisomerase 2	458.2466	3	0.4294	KTQMAEVLPSPR	43
C9J4C3	DNA topoisomerase 2	908.4190	3	-0.2327	LDDANDAGGRNSTECTLILTEGDSAK	120
C9J4C3	DNA topoisomerase 2	375.5318	3	0.3545	LHGGKDSASPR	78
C9J4C3	DNA topoisomerase 2	475.2696	3	-0.3213	LLFPPKDDHTLK	50
C9J4C3	DNA topoisomerase 2	395.5714	3	-0.0518	LSNKELKPQK	79
C9J4C3	DNA topoisomerase 2	800.7469	3	-0.5023	MEVSPLQPVNENMQVNKIKK	41
C9J4C3	DNA topoisomerase 2	845.7368	3	-0.7346	MYVPALIFGQLLTSSNYDDDEK	105
C9J4C3	DNA topoisomerase 2	883.1035	3	-0.3986	MYVPALIFGQLLTSSNYDDDEKK	61
C9J4C3	DNA topoisomerase 2	784.1432	4	-0.6536	MYVPALIFGQLLTSSNYDDDEKKVTGGR	142
C9J4C3	DNA topoisomerase 2	494.9072	3	0.4303	NYEDEDSLKTLR	76
C9J4C3	DNA topoisomerase 2	475.2562	3	0.3585	RAYDIAGSTKDVK	111
C9J4C3	DNA topoisomerase 2	593.9831	3	0.1333	RDPALNSGVSQKPDPAK	66
C9J4C3	DNA topoisomerase 2	636.6462	3	-1.0086	RLMDGEEPLPMLPSYK	50
C9J4C3	DNA topoisomerase 2	500.9328	3	0.3133	SIPSMVDGLKPGQR	48
C9J4C3	DNA topoisomerase 2	709.6410	3	-0.2953	SKGESDDFHMDFDSAVAPR	76
C9J4C3	DNA topoisomerase 2	991.2443	4	0.0981	SVVSDLEADDVKGSVPLSSSPPATHFPDETEITNPVPK	44

C9J4C3	DNA topoisomerase 2	614.8214	2	0.8599	TQMAEVLPSPR	101
C9J4C3	DNA topoisomerase 2	804.7299	3	0.3575	TWTQTYKEQVLEPMLNGTEK	62
C9J4C3	DNA topoisomerase 2	397.9885	4	-0.1083	VFLNGNKLPVKGFR	88
C9J4C3	DNA topoisomerase 2	659.3472	2	0.3943	YIFTMLSSLAR	79
Q02880	DNA topoisomerase 2-beta	359.8732	3	-0.8253	FKAQTQLNK	52
Q02880	DNA topoisomerase 2-beta	414.2347	3	0.5676	GKPSSDTVPKPK	81
Q02880	DNA topoisomerase 2-beta	362.8720	3	0.7570	GREVNDLKR	70
Q02880	DNA topoisomerase 2-beta	510.6169	3	-0.3885	HGFLEEFITPIVK	174
Q02880	DNA topoisomerase 2-beta	671.3344	4	-0.7738	HLTYNDFINKELILFSNSDNER	134
Q02880	DNA topoisomerase 2-beta	680.3894	3	-0.2464	HVDYVVDQVVGKLIEVVK	91
Q02880	DNA topoisomerase 2-beta	582.6442	3	-0.1266	IFDEILVNAADNKQR	123
Q02880	DNA topoisomerase 2-beta	389.5595	3	-0.0822	ILNVREASHK	55
Q02880	DNA topoisomerase 2-beta	979.7978	3	-1.0993	KTSFDQDSDVDIFPSDFPTEPPSLPR	90
Q02880	DNA topoisomerase 2-beta	760.8743	2	0.0562	LQLEETMPSPYGR	66
Q02880	DNA topoisomerase 2-beta	500.9438	3	0.2491	SIPSLVDGFKPGQR	62
Q02880	DNA topoisomerase 2-beta	466.5684	3	-0.3526	SYDDAESLKTLR	53
Q02880	DNA topoisomerase 2-beta	829.7478	3	-0.8241	VYVPALIFGQLLTSSNYDDDEK	48
Q02880	DNA topoisomerase 2-beta	872.4462	3	-0.7687	VYVPALIFGQLLTSSNYDDDEKK	66
Q02880	DNA topoisomerase 2-beta	772.1515	4	0.2253	VYVPALIFGQLLTSSNYDDDEKKVTGGR	94
P62807	Histone H2B type 1-C/E/F/G/I	592.6079	3	0.0268	AMGIMNSFVNDIFER	189
P62807	Histone H2B type 1-C/E/F/G/I	820.7264	3	0.9082	AMGIMNSFVNDIFERIAGEASR	96
P62807	Histone H2B type 1-C/E/F/G/I	328.8502	3	0.2151	AVTKYTSSK	75
P62807	Histone H2B type 1-C/E/F/G/I	584.3524	3	-0.4233	EIQTAVRLLLPGELAK	159

P62807	Histone H2B type 1-C/E/F/G/I	414.7141	2	-0.0110	HAVSEGTK	104
P62807	Histone H2B type 1-C/E/F/G/I	409.8946	3	-0.1258	HAVSEGTKAVTK	175
P62807	Histone H2B type 1-C/E/F/G/I	598.6513	3	-0.1595	HAVSEGTKAVTKYTSSK	182
P62807	Histone H2B type 1-C/E/F/G/I	529.2901	3	0.1882	IAGEASRLAHYNKR	115
P62807	Histone H2B type 1-C/E/F/G/I	422.5519	3	0.0435	KESYSVYVYK	89
P62807	Histone H2B type 1-C/E/F/G/I	535.9677	3	0.0880	KESYSVYVYKVLK	167
P62807	Histone H2B type 1-C/E/F/G/I	477.3051	2	-0.0812	LLLPGELAK	71
P62807	Histone H2B type 1-C/E/F/G/I	441.5070	4	-0.1314	LLLPGELAKHAVSEGTK	76
P62807	Histone H2B type 1-C/E/F/G/I	364.8731	3	-0.4260	PEPAKSAPAPK	136
P62807	Histone H2B type 1-C/E/F/G/I	390.2034	3	-0.0487	QVHPDTGISSK	90
P62807	Histone H2B type 1-C/E/F/G/I	539.9726	3	1.2034	RSTITSREIQTAVR	104
P62807	Histone H2B type 1-C/E/F/G/I	503.5963	3	-0.3514	SRKESYSVYVYK	199
P62807	Histone H2B type 1-C/E/F/G/I	487.9388	3	0.1818	STITSREIQTAVR	122
P62807	Histone H2B type 1-C/E/F/G/I	599.8522	4	-0.7458	STITSREIQTAVRLLLPGELAK	77
P62807	Histone H2B type 1-C/E/F/G/I	503.6192	3	0.0631	VLKQVHPDTGISSK	121
P36776	Lon protease homolog, mitochondrial	397.8728	3	-0.0751	ALCGLDESKAK	100
P36776	Lon protease homolog, mitochondrial	356.8577	3	0.1272	ALNREYFR	66
P36776	Lon protease homolog, mitochondrial	1291.6337	3	0.0568	GYQGDPSSALLELLDPEQNANFLDHYLDVPVDLSK	78
P36776	Lon protease homolog, mitochondrial	775.6318	4	0.7393	HVMDVVDEELSKLGLLDNHSSEFNVTR	66
P36776	Lon protease homolog, mitochondrial	351.8937	3	-0.0234	ILPVGGIKEK	80
P36776	Lon protease homolog, mitochondrial	1059.2101	3	0.4965	IVSGEAESVEVTPENLQDFVGKPVFTVER	105
P36776	Lon protease homolog, mitochondrial	500.6279	3	-0.4744	LAIAERYLVPQAR	88
P36776	Lon protease homolog, mitochondrial	464.2784	3	-0.4114	LAQPYVGVFLKR	98

P36776	Lon protease homolog, mitochondrial	603.6695	3	-0.6243	LSSDVLTLLIKQYCR	116
P36776	Lon protease homolog, mitochondrial	796.9090	2	0.5307	NYLDWLTSIPWGK	78
P36776	Lon protease homolog, mitochondrial	923.4608	3	0.3294	NYLDWLTSIPWGKYSNENLDLAR	113
P36776	Lon protease homolog, mitochondrial	540.5236	4	-0.3397	QLEVEPEEPEAENKHKPR	74
P36776	Lon protease homolog, mitochondrial	833.3910	5	0.8011	RDDSNESDVVESLDEIYHTGTFAQIHEMQDLGDKLR	52
P36776	Lon protease homolog, mitochondrial	906.9663	4	0.3790	SAYKIVSGEAESVEVTPENLQDFVGKPVFTVER	54
P36776	Lon protease homolog, mitochondrial	609.0070	3	-0.5357	TENPLILIDEVDKIGR	118
P36776	Lon protease homolog, mitochondrial	737.8916	4	-1.0571	TIRDIIALNPLYRESVLQMMQAGQR	101
P36776	Lon protease homolog, mitochondrial	685.3879	3	0.0298	TKTENPLILIDEVDKIGR	139
P36776	Lon protease homolog, mitochondrial	1228.9365	3	0.5697	VVDNPIYLSDMGAALTGAESHELQDVLEETNIPK	117
P36776	Lon protease homolog, mitochondrial	849.8335	5	0.0203	VVDNPIYLSDMGAALTGAESHELQDVLEETNIPKRLYK	48
P36776	Lon protease homolog, mitochondrial	597.7911	2	0.3798	YSNENLDLAR	77
Q14676	Mediator of DNA damage checkpoint protein 1	980.8607	3	0.4982	AAESLTAIPEPASPQLLETPIHASQIQK	36
Q14676	Mediator of DNA damage checkpoint protein 1	888.2282	4	-0.1156	AAESLTAIPEPASPQLLETPIHASQIQKVEPAGR	39
Q14676	Mediator of DNA damage checkpoint protein 1	600.3101	4	-0.5916	AAGNPGSLAAPIDHKPCSAPLEPK	42
Q14676	Mediator of DNA damage checkpoint protein 1	514.2499	3	0.0060	DQAEEEPNRIPSR	88
Q14676	Mediator of DNA damage checkpoint protein 1	748.0326	3	0.3126	LEPSTSTDQPVTPEPTSQATR	52
Q14676	Mediator of DNA damage checkpoint protein 1	852.1740	4	-0.7820	MTPFPATSAAPEPHPSTSTAQPVTPKPTSQATR	38
Q14676	Mediator of DNA damage checkpoint protein 1	934.7285	4	0.2094	SSGKTPETLVPTAPKLEPSTSTDQPVTPEPTSQATR	88
Q14676	Mediator of DNA damage checkpoint protein 1	756.8936	4	-0.1103	SSVKTPEPVVPTAPEPHPTTSTDQPVTPK	50
Q14676	Mediator of DNA damage checkpoint protein 1	1145.2493	3	0.1649	SSVKTPETVVPAAPELQPSTSTDQPVTPEPTSR	75
Q14676	Mediator of DNA damage checkpoint protein 1	950.2405	4	-0.1154	SSVKTPETVVPTALELQPSTSTDRPVTSEPTSQATR	82
Q14676	Mediator of DNA damage checkpoint protein 1	1133.2372	3	0.0006	SSVKTPETVVPTAPELQASASTDQPVTSEPTSR	82

Q14676	Mediator of DNA damage checkpoint protein 1	1126.2300	3	-0.3539	TPEPVVPTAPELQPSTSTDQPVTSEPTSQVTR	79
Q14676	Mediator of DNA damage checkpoint protein 1	875.1132	3	-0.0843	TPEPVVPTAPEPHPTTSTDQPVTPK	105
Q14676	Mediator of DNA damage checkpoint protein 1	1011.5068	3	-0.9907	TPETVVPAAPELQPSTSTDQPVTPEPTSR	62
Q14676	Mediator of DNA damage checkpoint protein 1	999.4947	3	0.0003	TPETVVPTAPELQASASTDQPVTSEPTSR	86
Q14676	Mediator of DNA damage checkpoint protein 1	1026.5382	3	-0.0080	TPETVVPTAPELQISTSTDQPVTPKPTSR	104
Q14676	Mediator of DNA damage checkpoint protein 1	1165.2493	3	-0.1516	TPKPVEPAASDLEPFTPTDQSVTPEAIAQGGQSK	74
P19338	Nucleolin, isoform 1	348.1929	3	0.1356	AGKNQGDPKK	133
P19338	Nucleolin, isoform 1	384.8998	3	-0.2113	AIRLELQGPR	138
P19338	Nucleolin, isoform 1	578.9956	3	0.6816	AIRLELQGPRGSPNAR	104
P19338	Nucleolin, isoform 1	544.6504	3	-0.2532	ALELTGLKVFGNEIK	166
P19338	Nucleolin, isoform 1	498.3118	3	-0.1267	ALVATPGKKGAAIPAK	47
P19338	Nucleolin, isoform 1	482.2684	3	0.1004	ATFIKVPQNQNGK	80
P19338	Nucleolin, isoform 1	438.2576	3	-0.2129	AVTTPGKKGATPGK	73
P19338	Nucleolin, isoform 1	782.7143	3	0.6047	EAMEDGEIDGNKVTLDWAKPK	111
P19338	Nucleolin, isoform 1	816.1696	4	-0.1778	FGYVDFESAEDLEKALELTGLKVFGNEIK	41
P19338	Nucleolin, isoform 1	465.9488	3	0.2718	GATPGKALVATPGKK	82
P19338	Nucleolin, isoform 1	781.3439	2	0.2731	GFGFVDFNSEEDAK	130
P19338	Nucleolin, isoform 1	611.2881	3	-0.1322	GFGFVDFNSEEDAKAAK	87
P19338	Nucleolin, isoform 1	734.0132	3	0.5886	GLSEDTTEETLKESFDGSVR	158
P19338	Nucleolin, isoform 1	809.7260	3	0.8582	GLSEDTTEETLKESFDGSVRAR	95
P19338	Nucleolin, isoform 1	700.3228	3	0.0594	GQNQDYRGGKNSTWSGESK	60
P19338	Nucleolin, isoform 1	667.5682	4	-0.2765	GYAFIEFASFEDAKEALNSCNKR	95
P19338	Nucleolin, isoform 1	912.4314	3	-0.2406	IVTDRETGSSKGFGFVDFNSEEDAK	143

P19338	Nucleolin, isoform 1	592.9491	3	0.9760	KFGYVDFESAEDLEK	119
P19338	Nucleolin, isoform 1	651.3376	4	-0.7448	KFGYVDFESAEDLEKALELTGLK	105
P19338	Nucleolin, isoform 1	337.8818	3	0.2470	KGAAIPAKGAK	103
P19338	Nucleolin, isoform 1	899.4729	3	0.5416	KQKVEGTEPTTAFNLFVGNLNFNK	140
P19338	Nucleolin, isoform 1	338.2220	3	-0.5283	KVAVATPAKK	111
P19338	Nucleolin, isoform 1	329.2183	3	-0.6655	KVVVSPTKK	109
P19338	Nucleolin, isoform 1	1024.4361	4	-0.8873	MAPPPKEVEEDSEDEEMSEDEEDDSSGEEVVIPQKK	73
P19338	Nucleolin, isoform 1	869.7815	3	0.1240	NLPYKVTQDELKEVFEDAAEIR	135
P19338	Nucleolin, isoform 1	1159.9018	3	0.0594	NSTWSGESKTLVLSNLSYSATEETLQEVFEK	159
P19338	Nucleolin, isoform 1	673.0006	3	-0.3923	QGTEIDGRSISLYYTGEK	40
P19338	Nucleolin, isoform 1	856.7746	3	-0.2439	QKVEGTEPTTAFNLFVGNLNFNK	101
P19338	Nucleolin, isoform 1	799.1687	4	-0.4634	QKVEGTEPTTAFNLFVGNLNFNKSAPELK	41
P19338	Nucleolin, isoform 1	848.7938	3	0.7305	SAPELKTGISDVFAKNDLAVVDVR	157
P19338	Nucleolin, isoform 1	674.6570	3	-0.3313	SISLYYTGEKGQNQDYR	136
P19338	Nucleolin, isoform 1	755.3696	3	-0.2550	SISLYYTGEKGQNQDYRGGK	125
P19338	Nucleolin, isoform 1	378.8888	3	-0.0213	SQPSKTLFVK	71
P19338	Nucleolin, isoform 1	503.9108	3	-0.2130	TFEEKQGTEIDGR	124
P19338	Nucleolin, isoform 1	640.3459	3	0.4741	TGISDVFAKNDLAVVDVR	146
P19338	Nucleolin, isoform 1	826.4442	3	0.2521	TGISDVFAKNDLAVVDVRIGMTR	50
P19338	Nucleolin, isoform 1	930.1344	3	-0.1070	TLFVKGLSEDTTEETLKESFDGSVR	136
P19338	Nucleolin, isoform 1	834.4268	3	-0.3273	TLVLSNLSYSATEETLQEVFEK	232
P19338	Nucleolin, isoform 1	1021.2042	3	-0.2678	TLVLSNLSYSATEETLQEVFEKATFIK	80
P19338	Nucleolin, isoform 1	982.5151	4	-0.8613	TLVLSNLSYSATEETLQEVFEKATFIKVPQNQNGK	61

P19338	Nucleolin, isoform 1	771.3901	3	0.5138	VEGTEPTTAFNLFVGNLNFNK	259
P19338	Nucleolin, isoform 1	979.8379	3	-0.5022	VEGTEPTTAFNLFVGNLNFNKSAPELK	78
P19338	Nucleolin, isoform 1	351.2080	4	0.2442	VFGNEIKLEKPK	72
P19338	Nucleolin, isoform 1	664.6689	3	0.2744	VTQDELKEVFEDAAEIR	148
P19338	Nucleolin, isoform 1	807.0954	3	0.9731	VTQDELKEVFEDAAEIRLVSK	129
P13010	Protein X-ray repair cross-complementing 5	321.8468	3	-0.3177	AFREEAIK	53
P13010	Protein X-ray repair cross-complementing 5	807.6841	4	0.2029	ALHPREPLPPIQQHIWNMLNPPAEVTTK	76
P13010	Protein X-ray repair cross-complementing 5	1106.1871	3	0.6769	ASFEEASNQLINHIEQFLDTNETPYFMK	154
P13010	Protein X-ray repair cross-complementing 5	780.1238	4	0.6511	CDISLQFFLPFSLGKEDGSGDRGDGPFR	36
P13010	Protein X-ray repair cross-complementing 5	913.8005	3	-0.7129	DDEAAAVALSSLIHALDDLDMVAIVR	107
P13010	Protein X-ray repair cross-complementing 5	842.3794	3	-0.0605	DKPSGDTAAVFEEGGDVDDLLDMI	88
P13010	Protein X-ray repair cross-complementing 5	748.3452	3	-0.3412	DQVTAQEIFQDNHEDGPTAK	82
P13010	Protein X-ray repair cross-complementing 5	885.1317	3	0.5489	EPLPPIQQHIWNMLNPPAEVTTK	55
P13010	Protein X-ray repair cross-complementing 5	967.7868	3	0.2773	ETVYCLNDDDETEVLKEDIIQGFR	58
P13010	Protein X-ray repair cross-complementing 5	1027.8251	3	-0.1540	FLAPKDKPSGDTAAVFEEGGDVDDLLDMI	100
P13010	Protein X-ray repair cross-complementing 5	506.6101	3	0.0296	FQRLFQCLLHR	192
P13010	Protein X-ray repair cross-complementing 5	524.6262	3	-0.1383	GITEQQKEGLEIVK	49
P13010	Protein X-ray repair cross-complementing 5	560.6299	3	-0.1422	HIEIFTDLSSRFSK	80
P13010	Protein X-ray repair cross-complementing 5	644.3204	3	0.0887	HLMLPDFDLLEDIESK	180
P13010	Protein X-ray repair cross-complementing 5	431.2505	3	-0.0060	IAAYKSILQER	122
P13010	Protein X-ray repair cross-complementing 5	424.9357	3	-0.3596	IKTLFPLIEAK	135
P13010	Protein X-ray repair cross-complementing 5	865.9146	4	-0.2009	KASFEEASNQLINHIEQFLDTNETPYFMK	56
P13010	Protein X-ray repair cross-complementing 5	627.6544	3	0.1370	KDEKTDTLEDLFPTTK	121

P13010	Protein X-ray repair cross-complementing 5	833.7419	3	-0.1027	KKDQVTAQEIFQDNHEDGPTAK	189
P13010	Protein X-ray repair cross-complementing 5	873.4445	4	0.7922	KLKTEQGGAHFSVSSLAEGSVTSVGSVNPAENFR	52
P13010	Protein X-ray repair cross-complementing 5	822.4246	3	-0.1309	KYAPTEAQLNAVDALIDSMSLAK	134
P13010	Protein X-ray repair cross-complementing 5	362.8674	3	-0.0958	LFQCLLHR	115
P13010	Protein X-ray repair cross-complementing 5	370.5416	3	0.1109	LGGHGPSFPLK	106
P13010	Protein X-ray repair cross-complementing 5	888.1569	3	-0.1796	LGGHGPSFPLKGITEQQKEGLEIVK	99
P13010	Protein X-ray repair cross-complementing 5	841.4207	4	-0.2742	LKTEQGGAHFSVSSLAEGSVTSVGSVNPAENFR	55
P13010	Protein X-ray repair cross-complementing 5	1016.2514	4	-0.6479	QVFAENKDEIALVLFGTDGTDNPLSGGDQYQNITVHR	139
P13010	Protein X-ray repair cross-complementing 5	338.5500	3	-0.3166	SQIPLSKIK	51
P13010	Protein X-ray repair cross-complementing 5	653.3458	3	-0.0569	TDTLEDLFPTTKIPNPR	132
P13010	Protein X-ray repair cross-complementing 5	368.2204	3	-0.7047	TLKKEDIQK	116
P13010	Protein X-ray repair cross-complementing 5	817.6815	4	0.0599	VFAARDDEAAAVALSSLIHALDDLDMVAIVR	127
P13010	Protein X-ray repair cross-complementing 5	505.2786	2	0.1154	VITMFVQR	96
P13010	Protein X-ray repair cross-complementing 5	779.7263	3	0.2345	YAPTEAQLNAVDALIDSMSLAK	223
P13010	Protein X-ray repair cross-complementing 5	657.9838	3	0.0965	YGSDIVPFSKVDEEQMK	104
P46060	Ran GTPase-activating protein 1	878.7784	3	-0.9728	AIANLYGPLMALNHMVQQDYFPK	53
P46060	Ran GTPase-activating protein 1	859.4684	3	0.1677	ALAPLLLAFVTKPNSALESCSFAR	121
P46060	Ran GTPase-activating protein 1	601.3506	2	0.2345	HSLLQTLYKV	66
P46060	Ran GTPase-activating protein 1	1097.5659	3	1.1537	ILDPNTGEPAPVLSSPPPADVSTFLAFPSPEK	105
P46060	Ran GTPase-activating protein 1	1224.9890	3	0.6336	ILDPNTGEPAPVLSSPPPADVSTFLAFPSPEKLLR	68
P46060	Ran GTPase-activating protein 1	541.9895	3	0.0794	SKGAVAIADAIRGGLPK	80
P46060	Ran GTPase-activating protein 1	821.7708	3	-0.1952	SSVLIAQQTDTSDPEKVVSAFLK	102
P46060	Ran GTPase-activating protein 1	440.9139	3	0.2841	TQVAGGQLSFKGK	44

P46060	Ran GTPase-activating protein 1	446.5735	3	-0.0226	VSSVFKDEATVR	55
P43487	Ran-specific GTPase-activating protein	984.1847	4	-0.2676	DTHEDHDTSTENTDESNHDPQFEPIVSLPEQEIK	93
P43487	Ran-specific GTPase-activating protein	540.9266	3	-0.1036	FASENDLPEWKER	168
P43487	Ran-specific GTPase-activating protein	726.6869	3	-0.1946	FASENDLPEWKERGTGDVK	80
P43487	Ran-specific GTPase-activating protein	437.2348	3	0.3173	FLNAENAQKFK	83
P43487	Ran-specific GTPase-activating protein	613.2870	4	-0.0831	ICANHYITPMMELKPNAGSDR	49
P43487	Ran-specific GTPase-activating protein	510.0102	4	-0.2324	LFRFASENDLPEWKER	107
P43487	Ran-specific GTPase-activating protein	691.3221	2	0.2915	TLEEDEEELFK	101
P43487	Ran-specific GTPase-activating protein	556.9310	3	0.3946	TLEEDEEELFKMR	139
P23246	Splicing factor, proline- and glutamine-rich	426.2032	3	0.2319	AELDDTPMRGR	55
P23246	Splicing factor, proline- and glutamine-rich	426.2032	3	0.1462	AELDDTPMRGR	55
P23246	Splicing factor, proline- and glutamine-rich	581.9697	3	-0.3033	ALAEIAKAELDDTPMR	150
P23246	Splicing factor, proline- and glutamine-rich	658.3422	3	-0.2152	ALAEIAKAELDDTPMRGR	91
P23246	Splicing factor, proline- and glutamine-rich	491.6108	3	-0.3333	AVVIVDDRGRSTGK	61
P23246	Splicing factor, proline- and glutamine-rich	760.7196	3	-0.4043	CRLFVGNLPADITEDEFKR	103
P23246	Splicing factor, proline- and glutamine-rich	1199.6062	3	0.4770	CSEGVFLLTTTPRPVIVEPLEQLDDEDGLPEK	154
P23246	Splicing factor, proline- and glutamine-rich	1010.0251	4	-0.5049	CSEGVFLLTTTPRPVIVEPLEQLDDEDGLPEKLAQK	69
P23246	Splicing factor, proline- and glutamine-rich	588.2655	3	-0.0092	FAQHGTFEYEYSQR	121
P23246	Splicing factor, proline- and glutamine-rich	519.9945	4	0.0294	FAQHGTFEYEYSQRWK	84
P23246	Splicing factor, proline- and glutamine-rich	381.8805	3	3.1809	FATHAAALSVR	80
P23246	Splicing factor, proline- and glutamine-rich	941.7324	4	0.4736	FATHAAALSVRNLSPYVSNELLEEAFSQFGPIER	62
P23246	Splicing factor, proline- and glutamine-rich	671.3366	2	0.0895	FGQGGAGPVGGQGPR	74
P23246	Splicing factor, proline- and glutamine-rich	820.3926	3	-0.9705	FGQGGAGPVGGQGPRGMGPGTPAGYGR	86

P23246	Splicing factor, proline- and glutamine-rich	385.2170	3	-0.5874	GFGFIKLESR	96
P23246	Splicing factor, proline- and glutamine-rich	535.4616	5	-0.3647	GGRQHHPPYHQQHHQGPPPGGPGGR	79
P23246	Splicing factor, proline- and glutamine-rich	415.9032	3	-0.0279	GIVEFASKPAAR	82
P23246	Splicing factor, proline- and glutamine-rich	415.9032	3	-0.0675	GIVEFASKPAAR	82
P23246	Splicing factor, proline- and glutamine-rich	458.6015	3	-0.3023	GIVEFASKPAARK	56
P23246	Splicing factor, proline- and glutamine-rich	335.4437	4	-0.1112	GKGFGFIKLESR	71
P23246	Splicing factor, proline- and glutamine-rich	550.6334	3	0.2175	ISDSEGFKANLSLLR	161
P23246	Splicing factor, proline- and glutamine-rich	412.8850	3	-0.1042	LAQKNPMYQK	70
P23246	Splicing factor, proline- and glutamine-rich	655.3423	3	-0.2395	LFVGNLPADITEDEFKR	94
P23246	Splicing factor, proline- and glutamine-rich	606.5797	4	-0.0481	LFVGNLPADITEDEFKRLFAK	100
P23246	Splicing factor, proline- and glutamine-rich	593.3117	4	-0.7630	MPGGPKPGGGPGLSTPGGHPKPPHR	73
P23246	Splicing factor, proline- and glutamine-rich	880.4378	3	-0.1505	NLSPYVSNELLEEAFSQFGPIER	199
P23246	Splicing factor, proline- and glutamine-rich	601.5392	4	-0.2203	QHHPPYHQQHHQGPPPGGPGGR	53
P23246	Splicing factor, proline- and glutamine-rich	412.5546	3	-0.8042	RPGEKTYTQR	126
P23246	Splicing factor, proline- and glutamine-rich	531.5299	4	-0.7422	SEEKISDSEGFKANLSLLR	97
P23246	Splicing factor, proline- and glutamine-rich	626.3072	3	0.1167	SLDEMEKQQREQVEK	76
P23246	Splicing factor, proline- and glutamine-rich	405.4782	4	0.2990	STGKGIVEFASKPAAR	121
P23246	Splicing factor, proline- and glutamine-rich	576.6239	3	0.1482	TERFGQGGAGPVGGQGPR	105
P23246	Splicing factor, proline- and glutamine-rich	466.9370	3	0.3792	VRFATHAAALSVR	86
P23246	Splicing factor, proline- and glutamine-rich	479.9173	3	-0.6885	YGEPGEVFINKGK	128
Q13428	Treacle protein	558.3003	3	-0.3191	ISDGKKQEGPATQVSK	124
Q13428	Treacle protein	867.7716	3	-1.3755	TQPSSGVDSAVGTLPATSPQSTSVQAK	154
Q13428	Treacle protein	769.0910	3	-0.0804	AAISNKITSCIFQLLQEAGIK	131

Q13428	Treacle protein	645.3738	3	0.1560	AASVPVKGSLGQGTAPVLPGK	84
Q13428	Treacle protein	688.8092	2	-0.7972	GMGTVEGGDQSNPK	76
Q13428	Treacle protein	634.6411	3	-0.8727	GSLGSQGAKDEPEEELQK	40
Q13428	Treacle protein	523.9702	3	0.0532	GTISAPGKVVTAAAQAK	114
Q13428	Treacle protein	434.5735	3	0.1224	KAEEDAALQAKK	103
Q13428	Treacle protein	679.0174	3	1.3368	LASTNSSVLGADLPSSMKEK	66
Q13428	Treacle protein	573.2919	3	-0.2487	SPQVKPASTMGMGPLGK	105
Q13428	Treacle protein	465.7664	2	0.8273	TGPTVTQVK	68
Q13428	Treacle protein	441.8994	3	-0.1997	TNVVTMPTAHPR	105
Q13428	Treacle protein	519.6058	3	-1.0026	TSQVGAASAPAKESPR	48
